# Inconsistency in the items included in tools used in general health research and physical therapy to evaluate the methodological quality of randomized controlled trials: a descriptive analysis

**DOI:** 10.1186/1471-2288-13-116

**Published:** 2013-09-17

**Authors:** Susan Armijo-Olivo, Jorge Fuentes, Maria Ospina, Humam Saltaji, Lisa Hartling

**Affiliations:** 1 Postdoctoral Fellow, CLEAR (Connecting Leadership and Research) Outcomes Research Program, University of Alberta, 5-115A Edmonton Clinic Health Academy (ECHA), 11405 – 87 Avenue, Edmonton, Alberta T6G 1C9 Canada; 2 Faculty of Rehabilitation Medicine, Department of Physical Therapy, University of Alberta, 3-48 Corbett Hall, Edmonton T6G 2G4, Canada; 3 Department of Physical Therapy, Catholic University of Maule, Talca, Chile; 4 School of Public Health, University of Alberta, Institute of Health Economics, Edmonton, Alberta, Canada; 5 School of Dentistry, Faculty of Medicine and Dentistry, University of Alberta, Edmonton, Alberta, Canada; 6 Alberta Research Centre for Health Evidence, Department of Pediatrics, Faculty of Medicine and Dentistry, University of Alberta, Edmonton, Alberta, Canada

**Keywords:** Bias, Methodological quality, Quality assessment, Critical appraisal, Risk of bias, Quality of reporting

## Abstract

**Background:**

Assessing the risk of bias of randomized controlled trials (RCTs) is crucial to understand how biases affect treatment effect estimates. A number of tools have been developed to evaluate risk of bias of RCTs; however, it is unknown how these tools compare to each other in the items included. The main objective of this study was to describe which individual items are included in RCT quality tools used in general health and physical therapy (PT) research, and how these items compare to those of the Cochrane Risk of Bias (RoB) tool.

**Methods:**

We used comprehensive literature searches and a systematic approach to identify tools that evaluated the methodological quality or risk of bias of RCTs in general health and PT research. We extracted individual items from all quality tools. We calculated the frequency of quality items used across tools and compared them to those in the RoB tool. Comparisons were made between general health and PT quality tools using Chi-squared tests.

**Results:**

In addition to the RoB tool, 26 quality tools were identified, with 19 being used in general health and seven in PT research. The total number of quality items included in general health research tools was 130, compared with 48 items across PT tools and seven items in the RoB tool. The most frequently included items in general health research tools (14/19, 74%) were inclusion and exclusion criteria, and appropriate statistical analysis. In contrast, the most frequent items included in PT tools (86%, 6/7) were: baseline comparability, blinding of investigator/assessor, and use of intention-to-treat analysis. Key items of the RoB tool (sequence generation and allocation concealment) were included in 71% (5/7) of PT tools, and 63% (12/19) and 37% (7/19) of general health research tools, respectively.

**Conclusions:**

There is extensive item variation across tools that evaluate the risk of bias of RCTs in health research. Results call for an in-depth analysis of items that should be used to assess risk of bias of RCTs. Further empirical evidence on the use of individual items and the psychometric properties of risk of bias tools is needed.

## Background

Randomized controlled trials (RCTs), and systematic reviews (SRs) and meta-analyses of these trials, are considered the gold standard to evaluate the effectiveness of health care interventions. Results of these studies are crucial for informing the implementation of the best treatments to improve patient outcomes and the efficiency of the health care system. Evaluating the methodological quality of trials is an essential component of SRs as only the best available evidence should inform clinical and policy decisions. An accurate assessment of study quality is key for the synthesis and interpretation of results across studies to effectively guide health care [1].

The term “methodological quality” has evolved since its inception and involves the evaluation of the internal validity as well as the external validity of a given study [2,3]. Recently, The Cochrane Collaboration has lead a shift in the approach to quality assessment, in which the concept of trial quality is linked to the internal validity of the study, namely risk of bias [4]. However, there is still inconsistency among researchers on how study quality is defined, and several terms have been used interchangeably in the literature (i.e. quality assessment, methodological quality, risk of bias, critical appraisal, trial quality).

While the impact of trial bias on evidence synthesis has been largely recognized, the approaches to quality assessment have been inconsistent and controversial [5]. A wide variety of tools have been developed to evaluate RCT quality in different health areas.[5,6]; many of them have not been developed using scientifically rigorous methods nor have they been validated [5]. In addition, there is no agreement on the optimal tool to accurately assess trial quality. The use of different tools for evaluating the quality of primary research in SRs can lead to discrepancies and skewed interpretations of SR results [7-9] and ultimately impact recommendations for clinical care.

In 2008, The Cochrane Collaboration [10] introduced the Risk of Bias (RoB) tool as a way to address shortcomings associated with existing tools and methods for quality assessment in SRs. Individual RoB items were selected based on a growing body of empirical evidence quantifying the association between certain characteristics related to the conduct of the trial and estimates of treatment effects [11-15]. For example, there is evidence that inadequate allocation concealment or lack of double-blinding are likely to overestimate treatment effects by 18% and 9%, respectively [12,14,15].

In order to guide a proper assessment of study quality or risk of bias to inform decision-making, it is important to identify which items have been included in different tools and whether these items truly evaluate the likelihood of bias, as defined by The Cochrane Collaboration [10,16] and other criteria [17]. This would be an important contribution for evidence synthesis.

Most of the studies that have evaluated the use of tools for quality assessment of RCTs [5,6,18,19] have not exhaustively assessed how these tools compare to each other in terms of their individual items and whether their use varies across different areas of health care research. For example, a recent study [19] examined the characteristics and methods of reviews assessing the quality of RCTs. While substantial variation in the use of quality tools across reviews was identified, the study did not describe in detail which items were most frequently included in the tools. The present study was designed to refine the analysis of existing tools by conducting a more comprehensive search (i.e., no language restrictions, larger number of databases), describing the psychometric properties of the tools used in general health research, and comparing the items included in these tools with the Cochrane RoB tool.

We conducted a previous systematic review that described which tools have been used to evaluate the methodological quality of RCTs in physical therapy (PT) research [5]. RCTs conducted in the area of PT have unique characteristics compared with pharmacological trials. Because of the nature of PT treatments (e.g., manual therapy, exercises), RCTs assessing PT interventions are often complex [20], and diverse aspects of their design (e.g., type and intensity of therapy, standardized or individually tailored approaches, therapists’ skills and experience) are likely to affect study results. It is unknown whether the tools to assess the quality of RCTs in PT differ from those used in general health research in terms of the items and type of bias they aim to address. The present study was designed to expand and update the analysis of our previous review [5] on quality tools for evidence synthesis.

The main objective of the present study was to describe the frequency of individual items included in tools that assess RCT quality in general health and PT research, and how they compare to items included in the RoB tool [4]. Secondary objectives were to 1) determine the nature of items included in general health and PT quality tools (i.e., evaluation of “conduct” versus “reporting”); 2) report on the psychometric properties of quality tools that have been formally evaluated; 3) determine whether individual items in the tools relate to certain threats to validity or precision [10,16,17] and 4) quantify the number of citations per tool, as a measure of usage since each tool’s inception and after inception of the RoB tool.

## Methods

### *Design*: observational, descriptive study

#### Search strategy

An update of a previous SR [5] on quality assessment tools was carried out to identify scales and their items used in the assessment of RCT quality in health and PT research. The updated search strategy incorporated key words identified by Dechartres et al. [19], with searches conducted from January 1st, 2007 to June 10, 2013 in the following bibliographic databases: Medline, Embase, Cinahl, ISI Web of Science, EMB Reviews-Cochrane Central Register of Controlled Trials and Cochrane Library and Best Evidence, All EBM Reviews -CDSR, ACP journal Club, DARE, CCTR, Global health, and HealthSTAR. Key words used in the search were: tool, critical appraisal, critical appraisal review, appraisal of methodology, appraisal of research methodology, research design review, quality assessment, methodological quality tool, RoB (tool), randomized (randomised) controlled trial, and RCT. Additionally, we manually searched the bibliographies of potentially relevant papers. The search was not limited by language of publication. For a sample search strategy, see Additional file 1.

#### Criteria for inclusion of studies in the review

Studies were included if they described or used a newly developed tool to evaluate the methodological quality/RoB of RCTs in any area of medical/health research and described any of its psychometric properties (i.e. validity, reliability, responsiveness). We excluded studies in which quality tools were developed for only one specific SR, studies that were not related to the development or psychometric testing of quality tools, and studies on generic tools that evaluated different types of research design (e.g., qualitative and quantitative studies). In addition, studies using modifications of existing tools were not considered for inclusion as they were likely not systematically developed. The RoB tool [4,10] was known to be newly developed after our previous SR (2008), and was included prior to the updated search; however, we searched for manuscripts reporting psychometric properties of the RoB tool.

#### Data screening

Two reviewers independently screened abstracts and titles obtained from the database searches. The full text of potentially relevant articles was retrieved for further assessment. Disagreements were resolved by consensus.

#### Data extraction

Data extraction was conducted in two phases. First, two researchers independently extracted information on content, construction, special features (e.g. area of development-clinical area-, number of items, selection of items for inclusion, time to complete, scoring instructions), and psychometric properties of the new tools. Information on face, content, construct, and concurrent validity, internal consistency, and reproducibility (intra and inter-rater reliability/agreement) was extracted. For this update, authors of original studies were not contacted to obtain additional information. The definitions of psychometric properties from Streiner and Norman [21-23] were used in the present study. Guidelines developed by Terwee et al. [24] were used to define quality of measurement properties. Briefly, quality of measurement included internal (internal consistency, relevance of items and representativeness of items of the scale-content validity) as well as external components of validity (the relationship with other tests in a manner that is consistent with theoretically derived hypotheses-construct validity). Intra and inter-rater reliability (i.e. repeatability of measurements taken by the same tester at different times and repeatability of measurements taken by different testers, respectively) were also considered. Definitions of psychometric properties for this review are provided in Additional file 2.

Second, two researchers independently extracted information on individual items used in the tools and the frequency of items across tools. Tools were categorized as relevant to PT if the authors specifically stated that the scale was developed for PT research, it was developed by a PT group, or if, according to Scopus searches, the tool was used in at least 5 PT reviews. Otherwise the tool was considered a general health research tool. One of the tools commonly used in both general health and PT research is the Jadad scale. This tool was included in both categories.

Items from the quality tools were grouped according to nine content categories that have been previously described [5]: 1) introduction, objectives, and design; 2) patient selection (inclusion and exclusion criteria, description of study participants); 3) assignment, randomization, and allocation concealment; 4) blinding; 5) interventions; 6) attrition, follow up and protocol deviations; 7) outcomes; 8) statistical analysis; and 9) miscellaneous.

#### Classification of items

Methodological quality (conduct) and quality of reporting are two concepts that overlap to some degree; however, they relate to different aspects of study quality. We defined methodological quality as “the confidence that the trial design, conduct, and analysis has minimized or avoided biases in its treatment comparisons” [6] (e.g., allocation concealment was appropriate). We defined quality of reporting as authors providing “information about the design, conduct and analysis of the trial,” [6] (e.g., method for concealing allocation was reported). Two researchers independently classified individual items based on whether they evaluated “reporting” and/or “conduct” of the trial.

Classifying quality items is a complex task due to unclear description of items in the tools, lack of general agreement in bias definitions [25], and the need for empirical evidence linking these items to bias. Two researchers independently classified each item according to whether they potentially addressed threats to validity (i.e., selection bias, detection bias, performance bias, attrition bias) or precision (Additional file 3). These categorizations have been used in other relevant sentinel work [17,26-28]. Items that dealt with several threats to validity were classified as addressing multiple biases [29]. Reviewers considered each item by asking “What type of threats to validity or precision are addressed by a given item?” or “What do authors intend to capture with a given quality assessment item?” Thus, items were classified into the threats to validity or precision that best represented the concepts being addressed. We performed this task in duplicate and based on the guidelines established. The same type of analysis has been conducted previously for prognosis research [28]. Disagreements in item classification were resolved by consensus.

#### Tool citation

Each quality tool was tracked in the Scopus database to determine the number of times that the tool was cited since its original paper/citation. The number of citations per tool was tracked from January 1, 2007 to July 4, 2013 to describe recent uses of the tool and to ascertain whether the use of the tool declined after introduction of the RoB tool. The RoB tool was originally described in Chapter 8 of the Cochrane Handbook [10]. Since books and book chapters are not indexed in electronic databases, it was more challenging to track citations for the RoB tool. We tracked RoB citations using Google Scholar and the journal publication by Higgins et al. [4], that reported on the RoB tool.

#### Analysis

Data were summarized descriptively as the frequency of each item across quality tools, and within general health and PT research. Comparisons of items from PT and general health research tools with the RoB tool were also conducted. Comparisons between the proportion of individual items used by PT tools and general health research tools were performed using Chi-squared or Fisher exact tests. The alpha level was set at α = 0.05. The level of agreement between reviewers for study selection and data extraction from quality tools was calculated using percentage agreement and the Kappa (κ) statistic [30]. Analyses were performed using Stata Statistical Software: Version 12, 2012 (College Station, TX: StataCorp LP).

## Results

The updated electronic searches identified 32,627 citations. Manual searches identified four additional studies based on their titles and abstracts. After screening titles and abstracts, 154 articles were deemed potentially relevant. The application of the selection criteria resulted in 148 excluded studies. The main reasons for exclusion of studies were: 1) the study used a quality tool for which information on construction, development and/or psychometric properties was not available (n = 40); 2) the tool was already included in the original review (n = 39); 3) the study used a tool that was not specific for RCT quality assessment (n = 23); 4) the study used a modified tool already included in the review (n = 20); 5) the study used an instrument that was not a quality tool (n = 11); 6) the study used a tool developed for the purposes of a single review (n = 8); 7) the study focused on animal research (n = 4); 8) the study did not focus on a particular tool (n = 2); and 9) information on the name of the tool was not provided (n = 1). A list of excluded studies and reasons for exclusion is available in Additional file 4. The level of agreement for study selection between reviewers was excellent (kappa = 0.96).

**Figure 1:**
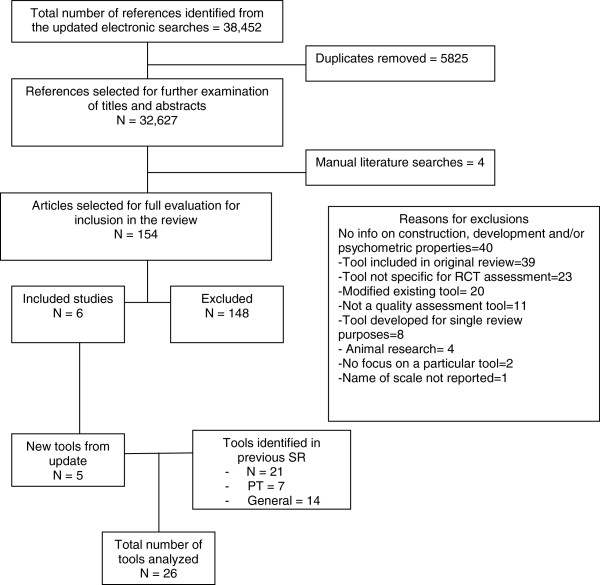
PRISMA flow diagram for identification of studies.

Six manuscripts [31-36] reporting on four newly developed tools met the eligibility criteria (Figure 1). These four new tools that evaluated the methodological quality/RoB of RCTs in health research in addition to the RoB tool [4,10] were: the Cochrane Collaboration Depression, Anxiety, And Neurosis (CCDAN) tool [34,35], the Randomized Controlled Trial Psychotherapy Quality Rating Scale (RCT-PQRS Tool) [32,33], 3) the Randomized Controlled Trial -Natural Products Tool (RCT-NP) [31], and the CLEAR NPT (a checklist to evaluate the report of nonpharmacological trials) [36]. New PT-specific tools were not identified. The five tools were added to the 21 tools identified in our previous review [2] (i.e. Jadad [37], Maastricht [38], Delphi [39], PeDro [40,41], Maastricht-Amsterdam [42], Van Tulder [43], Bizzini [44], Chalmers [45], Reisch [46], Andrew [47], Imperiale [48], Detsky [49], Cho and Bero [50], Balas [51], Sindhu [52], Downs and Black [53], Nguyen [54], Oxford Pain Validity Scale (OPVS) [55], Arrive [56], CONSORT [57], and Yates [58]). Therefore, this update includes 26 quality tools. Details on the characteristics and psychometric properties of the new quality tools are presented in Tables 1 and 2.\

**Table 1:** Characteristics of tools identified in the search update

***Study (authors, year)***	***Area***	***Numbers of items***	***How items were selected for inclusion***	***Validity***	***Reliability***	***Time to complete***	***Guidelines for use available***
**NEW TOOLS (2007-2013)**							
**COCHRANE COLLABORATION DEPRESSION, ANXIETY, AND NEUROSIS (CCDAN) [**[34,35]**]**	Trials of depression, anxiety and neurosis. Psychological and Psychiatric trials	23 items	This tool was developed from items included in other health tools (especially CONSORT statement), and then a consensus from experts was performed to determine a pilot tool to be tested.	Face, content and construct validity	Reliability evaluated through correlation coefficient among 3 raters in total score was high. It ranged from r=0.75-0.86.	15-20 minutes	No
				Scores from raters correlated highly with year of publication (r=0.37-0.6)			
					Reliability for individual items was less strong		
			Further validation consisted on determine reliability of the tool as well as internal consistency and its correlation with overall score and year of publication.		The mean kappa for all 23 items ranged between 0.51 to 0.54 among 3 raters		
					Internal consistency measured through Cronbach alpha ranged between 0.65 to 0.78		
**THE RANDOMIZED CONTROLLED TRIAL PSYCHOTHERAPY QUALITY RATING SCALE (RCT-PQRS TOOL) [**[32,33]**]**	Psychotherapy	25 items organized in 6 domains: Description of subject (4 items), definition and delivery of treatment (5 items), outcome measures (5 items), data analysis (5 items), treatment assignment (3 items), overall quality of study (3 items)	Items were generated by an informal expert consensus (members of the American Psychiatric Committee on Research on Psychiatric treatments, outside consultants, who were senior psychotherapy and/or psychopharmacology clinical researchers.	The Cronbach α for all 25 items as rated by the primary rater was 0.87.	The ICC for interrater reliability of item 25, the omnibus rating of the quality of the study, was 0.79.	10-15 minutes	Yes
				The correlation between the 24-item total and the omnibus item (item 25) was 0.88.	The ICC for interrater reliability of the total of the first 24 items was 0.76.		
				The correlation between the 24-item total and study year was 0.51, significant at P < .0001.	Nine of the individual items had individual ICCs between 0.5 and 0.8 (items 2, 4, 6, 7, 8, 10, 14, 15, and 19).		
				The correlation of the omnibus item and study year was 0.47 (P < .0001).	Twelve items had individual ICCs between 0.3 and 0.5 (items 1, 3, 5, 9, 11, 12, 13, 16, 17, 18, 20, and 24), and 3 items had individual ICCs below 0.3 (items 21, 22, and 23).		
					Two items had very low variation between studies (77% of studies received a 0 on item 13 and 97% of studies received a 2 on item 21).		
**THE RCT-NATURAL PRODUCTS TOOL (RCT-NP) [**[31]**]**	Trials of Natural products	28 items	The initial list of items for this study was compiled from items contained in published critical appraisal instruments designed for RCTs of NPs as well as from items suggested by the research team.	Comparisons with a published instrument to evaluate the methodological quality of RCTs for Natural product was used (criterion validity). Similar results were obtained with both instruments indicating criterion validity (Concurrent validity)	Not reported	Not reported	Yes
			A Delphi process was used to achieve consensus among a group of experts as to which items describing the identity of an NP were essential to consider when critically appraising an RCT of an NP.				
				Raters’ answers were compared with investigators answers to determine criterion validity as well. No significant differences between raters and investigators (gold standard) answers were obtained			
			The consensus building process was conducted in 2 rounds using email.				
			Consensus was considered to have been reached when 80% of participants were in agreement with an item being designated as essential to include in the instrument				
			A final list of items considered to be essential by the study participants and investigators was assembled.				
			A systematic review regarding tools used in to evaluate quality of NPs trials was performed. Items from all of these tools were compiled				
			To be designated as essential to include in the new critical appraisal instrument, an item had to meet at least 1 of the following 2 inclusion criteria: it had to have been contained in a published instrument that was documented as having been validated or must have had empirical evidence to support its inclusion in a published instrument.				
**A CHECKLIST TO EVALUATE A REPORT OF A NONPHARMACOLOGICAL TRIAL (CLEAR NPT) [**[51]**]**	Health Research	10 items and 5 subitems	Initial pool of items was performed from existing quality tools identified by Moher et al. and Verhagen and the CONSORT statement, users’ guides to the medical literature, and the Cochrane Reviewers’ Handbook.	Content validity was provided by experts in the field through the Delphi method	Not reported	10 minutes	Yes
			Items specific to NPT trials identified in a preliminary study and during informal interviews of clinicians working in the field of NPT were added.				
			Thirty-eight potential items were identified.				
			A Delphi procedure was used to determine the final items included in the tool.				
**RISK OF BIAS TOOL (RoB) [**[4,10]**]**	Health Research	The risk of bias tool is based on six domains and 7 items: sequence generation, allocation concealment, blinding, incomplete outcome data, selective outcome reporting, and “other sources of bias.” Critical assessments on the risk of bias (high, low, unclear) are made separately for each domain.	The choice of components for inclusion in the tool was based on empirical evidence showing their association with effect estimates.	Content validity: items were included based on empirical evidence.	Interrater agreement for the individual domains of the risk of bias tool ranged from slight (κ=0.13 for selective reporting) to substantial (κ=0.74 for sequence generation [13].	~21 minutes	Yes
				Concurrent validity: A high degree of correlation was found between the domains of risk of bias sequence generation compared with Jadad randomisation (k=0.79) and risk of bias allocation concealment compared with Schulz allocation concealment (k=0.73) [13]			
					The RoB demonstrated moderate to substantial (mean values 0.56 to 0.76) agreement on three of twelve items [59].		
					The interrater agreement was fair (0.40) for selective outcome reporting and almost perfect (0.86) for sequence generation [62].		
				Correlation was low for the comparisons between the domains of risk of bias incomplete outcome data and the Jadad withdrawal item, risk of bias overall risk and total Jadad score, and risk of bias overall risk and Schulz allocation concealment [13]			
					Interrater agreement for the majority of domains and overall risk of bias was moderate (k = 0.41–0.60) [60].		
				The correlations between overall risk of bias assessments and total Jadad score (t= 0.04) and allocation concealment (t = 0.02) were low [60].	The inter-rater reliability across individual domains of the CCRBT was found to be 0.30, which is considered slight agreement between raters [46]. The inter-rater reliability of the final grade assigned to each paper by this tool was ICC = 0.58 (95% CI 0.20–0.81)[61]		
					There was very poor agreement between the Effective Public Health Practice Project Quality Assessment Tool (EPHPP) and the RoB tool in the final grade assigned to each study (kappa = 0.006)[61]		
					The inter-rater reliability was substantial for sequence generation (k=0.79) and fair for the other 5 items (k=0.24-0.37). Interrater reliability between consensus evaluations across rater pairs was fair for allocation concealment and “other sources of bias” (k=0.37-0.27), and moderate for sequence generation (k=0.60). [62]		

95% *CI* = 95% confidence interval, *CONSORT* Consolidated Standards of Reporting of Trials, *ICC* intraclass correlation coefficient, *k* kappa, *NP* natural products, *NPT* natural products trials, *RCT* randomized controlled trial, *RoB* risk of bias.

**Table 2:** Summary of the quality of measurement properties of quality tools from our previous systematic review and this update

**Scale**	**Internal consistency**	**Face validity**	**Content validity**	Criterion Validity*	**Construct validity**	**Reproducibility (agreement/reliability)**
**TOOLS FOR PT**						
Jadad Tool [37]	-	+	+	+	+	+
Maastricht Tool [38]	-	+	-	+	-	+
Delphi Tool [39]	-	+	+	+	-	+
PEDro Tool [40,41]	-	+	+	-	-	+
Maastricht-Amsterdam Tool [42]	-	+	+	-	-	+
Van Tulder Tool [43]	-	+	+	+	-	+
Bizzini tool [44]	-	+	+	-	-	+
**TOOLS FOR GENERAL HEALTH RESEARCH**						
**Tools from previous systematic review**						
Chalmers tool [45]	-	+	+	+	-	+
Reisch Tool [46]	-	+	-	+	-	-
Andrew tool [47]	-	+	-	-	-	+
Imperiale tool [48]	-	+	-	+	-	-
Detsky tool [49]	-	+	-	+	-	+
Cho Tool [50]	-	+	-	+	-	+
Balas tool [51]	-	+	-	-	-	-
Sindhu tool [52]	-	+	+	+	-	+
Downs and Black tool [53]	+	+	+	+	-	+
Nguyen tool [54]	-	+	-	-	-	-
Oxford pain validity tool [55]	-	+	-	-	-	-
Arrive tool [56]	-	+	-	-	-	+
CONSORT tool [57]	-	+	+	-	-	+
Yates Tool [58]	-	+	+	-	+	+
**New tools identified in search update**						
Cochrane Collaboration Depression, Anxiety, and Neurosis (CCDAN) [34,35]	+	+	+	-	+	+
The Randomized Controlled Trial Psychotherapy Quality Rating Tool (RCT-PQRS Tool) [32,33]	+	+	+	-	+	+
RCT-Natural Products Tool (RCT-NP) [31]	-	+	+	+	-	-
CLEAR NPT (Checklist to evaluate a report of a nonpharmacological trial [36]	-	+	+	-	-	-
Risk of Bias Tool (RoB) [4,10]	-	+	+	+	+	+

+Quality of measurements properties were based on guidelines established by Terwee et al. [13].

(+): criterion accomplished.

(−): Criterion not accomplished.

* In all cases, criterion validity was established with “no gold standard tools”.

*PT* physical therapy, *RCT* randomized controlled trial.

Most of the new tools have been tested for face and content validity (Table 2 and Additional file 5). Evaluations of other types of validity, such as criterion validity, have been conducted only for the RCT-NP and the RoB tool; however, the criterion used was a non-gold standard tool (since to date, there is no accepted gold standard to evaluate the risk of bias or quality of RCTs in health research). Tool reproducibility has been evaluated for the CCDAN tool, the RCT-PQRS, and the RoB tool. The inter-rater reliability of the RoB tool was fair (k = 0.41-0.60) in contrast to the CCDAN and RCT-PQRS tools which showed good inter-rater reliability (r = 0.75-0.86; intra class correlation coefficient [ICC] =0.76-0.79).

Items from all 26 tools were summarized according to their frequency of use. The level of agreement between reviewers for item categorization in both PT (kappa = 0.92) and general health research tools (kappa = 0.98) was very good to excellent.

### Tools to measure methodological quality/risk of bias

Of the 26 tools, 19 have been used in general health research and seven in PT research (including the Jadad scale, which is commonly used in both research areas). A total of 130 items have been used across general heath research tools compared with 48 items used in PT tools. The RoB tool has 6 domains with 7 items in total. Additional files 5 and 6 provide a detailed description of individual items contained in the tools. The numbers of quality items according to the nine content categories for general health versus PT tools were: introduction, objectives, and design: 8 versus 0 items; patient selection: 18 versus 4 items; assignment, randomization, and allocation concealment: 8 versus 5 items; blinding: 12 versus 10 items; interventions: 17 versus 8 items; attrition, follow up and protocol deviation: 10 versus 9 items; outcomes: 15 versus 7 items; statistical analysis: 31 versus 5 items, and miscellaneous: 11 versus 0 items.

### Frequency of items: General health research tools, physical therapy tools and RoB tool

Items addressing inclusion and exclusion criteria and the appropriateness of statistical analysis were the most frequently used among the general health research tools (74%, 14/19 tools). The second most commonly used items in general health research tools were description of withdrawals and drop outs, description and appropriateness of randomization process, blinding of investigators/assessors, and description of treatment protocol for both intervention and control groups (63%, 12/19 tools).

**Figure 2:**
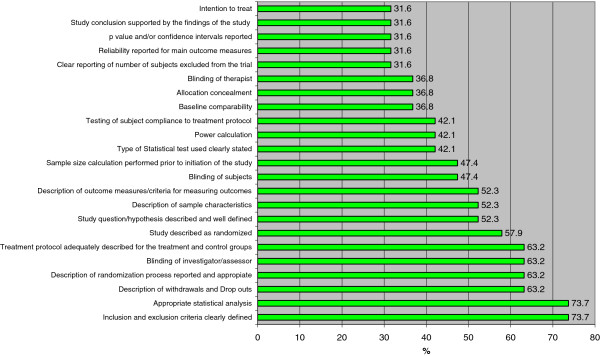
**Frequency of items used by tools used in general health research to measure methodological quality RCTs.** RCT = randomized controlled trial.

**Figure 3:**
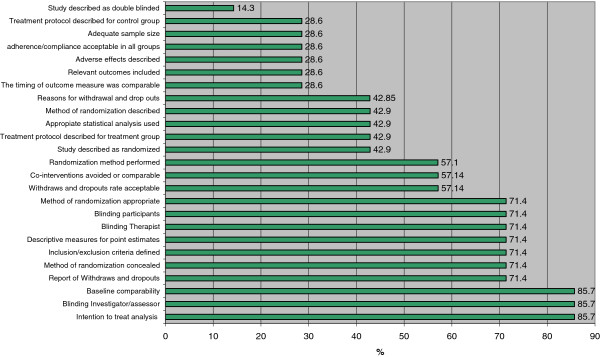
**Frequency of items used from tools used in PT research to measure methodological quality of RCTs.** PT = physical therapy; RCT = randomized controlled trial. Results expressed as percentages.

In contrast, baseline comparability, blinding of investigator/assessor, and use of intention-to-treat analysis were the most frequently used items among PT tools (86%, 6/7) (Additional files 5 and 6, Figures 2 and 3). The second most frequently used items in PT tools were: reporting of withdrawals and dropouts, method of randomization concealment, description of inclusion/exclusion, reporting of descriptive measures for point estimates, blinding of therapist, and blinding of participants (71%, 5/7 tools) (Figures 2 and 3).

Inclusion of the following items was significantly more frequent in PT tools compared with general health research tools: “intention to treat” (p = 0.014), “withdraws and drop outs acceptable” (p < 0.001), and “baseline comparability” (p = 0.027).

**Figure 4:**
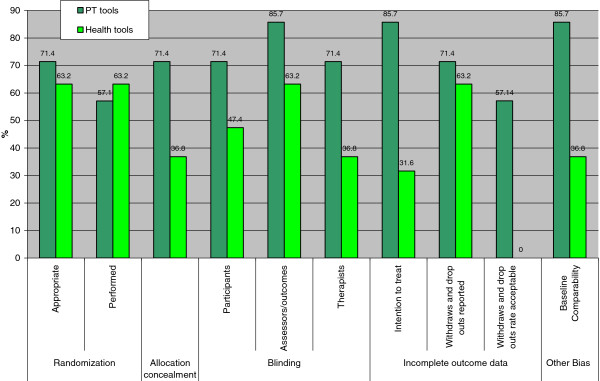
**Comparison between RoB tool domains and items from PT and health sciences tools.** PT = physical therapy; RoB = risk of bias. Results expressed as percentages.

When RoB items were individually examined, we found that sequence generation and allocation concealment were included in 5 of the 7 PT tools (Figure 4). Only four PT tools evaluated whether randomization was performed. Twelve (63%) general health research tools included randomization whereas seven (37%) included an item for allocation concealment. Further, fewer of the general health research tools included items related to blinding compared with the PT tools: blinding of participants (47% versus 71%) and blinding of outcome assessors (63% versus 86%). Intention to treat analysis, a component of the incomplete outcome data domain in the RoB tool was more frequently used in PT tools (86%) compared with general health research tools (32%). Other items related to incomplete outcome data in the RoB tool are “description of withdrawals and drop outs” and “appropriateness of withdrawal/drop outs rate”. Compared with the general health research tools (63%), a larger proportion of PT tools (71%) included items for the description of withdrawals and drop outs. In contrast, none of the general health research tools included an item about whether the withdrawal/drop-out rate was acceptable compared with 57% of the PT tools. Another quality item used in the RoB tool is baseline comparability. This item was included in 86% of PT tools compared with 37% of the general health research tools. In general, PT tools appeared more similar in content to the RoB tool than those used for general health research (Figure 4).

### Reporting versus conduct items and threats to validity and precision

Of the 130 items included in the general health research tools, 62 (48%) evaluated trial “reporting” whereas 60 items (46%) evaluated “conduct” (i.e. methodological quality or risk of bias). Eight items (6%) were considered to evaluate both quality of reporting and conduct of trials (i.e. sample representativeness and description of participants source, description of randomization process reported and appropriate; testing of subject compliance to treatment protocol /report of compliance; therapist training and level of experience in the treatment(s) under investigation; validity, reliability and responsiveness of the outcome measures reported; post-hoc power calculations and confidence intervals reported).

Classification of items from general health research tools according to type of bias and threats to precision was as follows: selection bias (25 items, 19%); performance bias (six items, 4.6%); performance and detection bias (six items, 4.6%); performance bias and contamination (seven items, 5.4%); performance bias and compliance (two items, 1.6%); attrition bias (ten items, 7.8%); information bias (six items, 4.6%); detection bias (five items, 3.8%); reporting bias (17 items, 13%); threats to precision (four items, 3.1%); statistical bias (three items, 2.3%); threats to precision and statistical bias (two items, 1.6%); multiple biases (three items, 2.3%); and other (i.e., not classified as related to threats to validity or precision) (27 items, 21%) (Additional file 5).

Of the 48 items included in PT tools, 16 (33.3%) evaluated trial “reporting” whereas 28 (58.3%) evaluated “conduct”. Four items (8.3%) were considered to evaluate both quality of trial reporting and conduct: testing/report subject compliance to treatment protocol, and validity, reliability and responsiveness of the outcome measures reported.

The classification of items from PT tools according to type of bias and threats to precision was as follows: selection bias (10 items, 21%); performance and detection bias (five items, 10%); performance bias only (six items, 135%); performance and contamination bias (two items, 4.2%); performance and compliance bias (two items, 4.2%); information bias (five items, 10%); attrition bias (eight items, 17%); detection bias (three items, 6.3%); reporting bias (three items, 6.3%); threats to precision (two items, 4.2%); and statistical bias (1 item, 2.1%). (Additional file 6).

### Frequency of citations of quality tools

The number of citations per quality tool since its inception and after 2007 are detailed in Table 3. The Jadad scale was, by far, the most cited and used tool with 5,326 citations from inception (i.e., year 1996) to July 4, 2013. The second most cited tool was the Downs and Black tool, with 962 citations since its introduction in 1998. Other tools frequently cited were: PeDro, Delphi, and Chalmers tools (651, 625, and 584 citations from inception, respectively), followed closely by the Van Tulder (560 citations) and Maastricht-Amsterdam (360 citations) tools. Among the most frequently cited tools, a larger proportion (5/7) were PT tools compared with only two of the 19 general health research tools (i.e. Jadad and Chalmers). The relative number of citations for the tools after 2008 was similar to those of previous years. Particularly, the use of the Jadad tool (i.e. number of citations from 2007 to July 4, 2013: 3,672) did not show a decrease (in terms of absolute numbers of citations) after the inception of the RoB tool in 2008 (Table 3). Tracking of the RoB tool showed that it has been cited approximately 1230 times since inception. This number is likely an underestimate because of the challenges described above with respect to tracking the Cochrane Handbook chapter that first described the tool. However, this information provides a reference point to track usage of the RoB tool over time.

**Table 3:** Frequency of citations of Quality Tools in Scopus Database

**Tool**	**All years until July 4, 2013**	**From January 2007-July 4, 2013**	**Year 2007 ** **before RoB tool**	**Year 2008**	**Year 2009**	**Year 2010**	**Year 2011**	**Year 2012**	**Year 2013 Until July 4, 2013**	**Subject area most used**
**PT TOOLS**										
Jadad [37]	5326	3672	393	468	616	514	634	706	341	Medicine
Maastricht [38]	106	45	3	6	10	5	7	9	5	Medicine/health professions
Delphi [39]	625	454	39	65	71	77	87	75	40	Medicine/health professions
PEDro [40,41]	651	555	49	52	87	91	101	108	67	Health professions/ Medicine
Maastricht-Amsterdam [42]	360	158	34	36	23	21	21	19	4	Medicine/health professions
Van Tulder [43]	560	482	43	58	101	86	65	93	34	Medicine/health professions
Bizzini [44]	65	50	4	15	7	6	9	4	5	Medicine/health professions
**GENERAL HEALTH RESEARCH TOOLS**										
**Tools From Previous Systematic Review**										
Chalmers [45]	584	151	19	21	29	19	19	29	15	Medicine/Psychology
Reisch [46]	56	26	2	4	5	2	8	4	1	Medicine/ Nursing
Andrew [47]	10	0	0	0	0	0	0	0	0	Medicine
Imperiale [48]	141	47	6	7	8	4	8	10	4	Medicine/ Pharmacology, Toxicology and Pharmaceutics
Detsky [49]	281	120	15	20	21	14	17	20	13	Medicine/ Biochemistry/ Nursing
Cho [50]	109	30	4	4	5	3	5	5	4	Medicine/ Pharmacology, Toxicology and Pharmaceutics
Balas [51]	37	5	0	3	1	0	0	0	1	Medicine
Sindhu [52]	31	11	2	2	3	1	3	0	0	Medicine/Nursing
Downs and Black [53]	962	783	80	72	100	125	146	163	97	Medicine/Nursing
Nguyen [54]	84	63	6	9	9	9	14	11	5	Dentistry/Medicine
Oxford pain validity tool [55]	143	49	11	14	7	10	2	3	2	Medicine/Neuroscience
Arrive [56]	23	6	2	2	2	0	0	0	0	Medicine
CONSORT [57]	184	124	30	21	20	14	14	17	8	Medicine/Biochemistry
Yates [58]	35	33	2	1	4	7	8	6	5	Medicine/Neuroscience
**NEW TOOLS**										
**Cochrane Collaboration Depression, Anxiety, and Neurosis (CCDAN) [**[34,35]**]**	59	44	3	6	11	6	10	7	1	Medicine/psychology
**The Randomized Controlled Trial Psychotherapy Quality Rating (RCT-PQRS) [**[32,33]**]**	30	30	0	0	0	0	10	16	4	Medicine/psychology
**RCT-Natural Products (RCT-NP) [**[31]**]**	3	3	0	0	1	1	1	0	0	Pharmacology, Toxicology and Pharmaceutics
**CLEAR NPT (Checklist to evaluate a report of a nonpharmacological trial [**[36]**]**	108	102	16	18	17	10	17	16	8	Medicine
**Risk of Bias (Scopus Track) Higgins et al., 2011 [**[4]**]**	124						1	41	75	Medicine
**Risk of Bias(Google Scholar) Chapter 8:Cochrane Handbook (2008-July4, 2013) [**[10]**]**	1155								1155	Medicine
**Total RoB**	1230*									

*This number could include duplicates; *RoB* risk of bias.

## Discussion

This study examined tools and individual items used in general health and PT research to assess the quality of RCTs. A variety of tools are still widely used despite criticisms raised regarding their limitations [8,63]. This finding is consistent with previous reviews on this topic that have identified inconsistencies in the use of quality tools [5,6,18]. There is extensive variation in individual items included across quality assessment tools. Many of these items may not be indicators of bias nor related to over- or under-estimations of treatment effects. Moreover, there is lack of empirical evidence supporting the association of many individual quality items with changes in the magnitude and direction of treatment effects. This finding raises important concerns in the field of quality assessment regarding the appropriateness of evaluating the evidence based on the use of these tools and items.

Results of this study agree with those of Deschartres et al. [19] which found that a large number of tools have been used in reviews that assessed the quality and reporting of RCTs. According to Deschartres et al. [19], ambiguity and lack of a unique definition of trial “quality” accounts for the heterogeneity of quality assessment tools. According to Verhagen et al. [2], methodological quality assessment involves the evaluation of internal validity (the degree to which the study design, conduct and analysis have minimized biases), external validity (the extent to which study results are generalizable beyond the experimental situation), and statistical analysis of primary research. According to The Cochrane Collaboration [10], internal validity of a trial is linked to “risk of bias” and it should be the primary focus of quality assessment since external validity differs upon context. In addition, “quality of reporting” is commonly used as a proxy for trial quality, which has complicated the construct of “quality” even more.

A clear and consistent definition of “quality” across health research areas is necessary to advance the field of quality assessment. Furthermore, concepts such as internal validity, external validity, and quality of reporting should be explicitly and clearly defined for the constructs that the individual items are meant to address. Finally, items assessing the methodological quality (or internal validity) of RCTs should be based on empirical evidence of their association with treatment effects.

The number of items across quality tools is large; 130 and 48 items have been used by tools in general health and PT research, respectively. Some items are subjective, confusing, and lack a clear definition (e.g., subjects appropriate to study questions*,* discussion of bias resulting from non-blinding assessment). These factors make the evaluation of individual items challenging and likely contribute to low inter-rater agreement. Many quality items relate to “reporting” rather than “conduct” of trials; approximately half of the items from these tools relate to reporting only. This finding is consistent with results described by Deschartres et al. [19], in which 25% of methodological reviews stated that RCTs reported details of sample size calculation, but only 6% reported on adequacy of the sample size. Although clear reporting is necessary to assess the quality of trial conduct, a focus on quality of reporting can hide differences in trial conduct and lead to under- or over-estimation of the methodological quality [64].

### Comparison of items between PT and general health research tools with RoB tool

We found that items frequently included in the PT tools were more closely linked to items/domains included in the RoB tool than those of general health research tools. This result suggests that PT tools are more closely linked to an examination of bias than the general health research tools.

Empirical evidence has supported many items in the RoB tool. There is a substantial interest in investigating which methodological features of RCTs are associated with treatment effects. Evidence informing this association comes mainly from RCTs in the area of medicine and is based primarily on evaluations of dichotomous outcomes [12,14,15]. Therefore, empirical evidence on the relationship between trial quality and treatment effects may not be readily applicable to other health research areas such as PT and other areas of rehabilitation. Morever, information regarding the importance of including certain items in quality tools within different clinical areas is limited. As mentioned previously, RCTs in the area of PT have distinct characteristics compared with pharmacological trials conducted in medicine. PT interventions are complex interventions [20]; they comprise certain characteristics such as the type of therapy and its intensity, a standardized or individually tailored approach, and the skills and experience of the therapists, that are likely to affect trial results. In addition, because of the nature of certain PT interventions (e.g., manual therapy, exercises), blinding of therapists and/or patients is not always possible. Appropriate blinding of study participants and all key study personnel is unlikely to be accomplished for most PT trials; however, blinding of outcome assessment has been commonly used as a proxy quality measure without validation. Therefore, more empirical evidence on trial bias is needed in the area of PT to determine which factors are likely to affect treatment effect estimates and thus provide accurate results for the clinical community. Further research should examine the appropriateness of using certain items/domains when evaluating the risk of bias of primary research in a variety of health areas. This information would provide clear benchmarks to assess the quality or risk of bias of primary research included in SRs and meta-analysis, and ultimately strengthen the evidence for decision-making in all areas of health care.

The RoB tool is recommended by The Cochrane Collaboration. Some groups within the Collaboration have developed their own tools and have not yet adopted the RoB approach (e.g. Cochrane Bone, Joint and Muscle Trauma Group). Other Cochrane groups have modified the RoB tool for their own purposes (i.e. Cochrane Back Review Group, Cochrane Renal Review Group). The RoB tool was developed more recently than many of the other tools; current research [9,13] recommends further testing of its psychometric properties and validation of the tool in a wide range of research fields. Additional guidelines will help users in applying and interpreting the results of the RoB tool.

### PT and general health research tool items and threats to validity and precision

Most items from general health research and PT tools were classified according to one or more categories of threats to validity or precision; however, some items could not be placed in any category. For example, the item “study question/hypothesis/purpose described and well defined” was not linked to any type of bias and was found irrelevant for study quality. Nevertheless, this item was included in 10 (53%) health research tools. This situation raises concerns about the usefulness of certain items to determine trial quality; therefore, these types of items should be carefully considered when deciding whether they should be part of these tools.

Classifying quality items was a complex task due to unclear descriptions of the items and lack of empirical evidence linking these items to bias. The number of items that was linked to different types of bias varied by tool. For example, a high percentage of items dealt with selection bias (approximately 19% of general health and 21% of PT tools). In contrast, attrition bias was more frequently represented in items found in PT (17%) compared with general health research (7%) tools. These results call for an in-depth analysis of individual items of tools that evaluate trial quality or risk of bias of RCTs in health research in order to provide a more complete assessment of their internal validity.

### Tools most cited

The Jadad scale [37] is the most frequently cited tool in health sciences research despite criticisms regarding its lack of responsiveness [8] and applicability to other health research areas such as PT and rehabilitation [5]. Herbison et al. [8], found that the Jadad scale might not be responsive enough to distinguish among different levels of trial quality. The use of the Jadad scale has been discouraged in many areas of health research. The discordance between recommendations against using the Jadad scale and its ongoing use is a matter of concern and reasons for this discrepancy should be further explored. It is likely that the Jadad tool is popular among SR authors because it is simple and requires little time to apply [13].

None of the other quality tools used in general health research and PT is as highly cited as the Jadad tool. Some tools are specific to certain areas (e.g., PT, nursing, psychology, pharmacology); most of them are long instruments and require a greater amount of time to complete; and some lack clear guidelines for item assessment, which can discourage their use.

### Strengths and limitations

To the best of our knowledge, this study is the first to exhaustively explore the type and frequency of individual items included in tools that evaluate the quality or risk of bias of RCTs in health research. A comprehensive search was performed for all published research in this area, with no language restrictions, and using several strategies (i.e., manual search, Scopus) to identify relevant literature. However, because of indexing problems of research on the evaluation of quality assessment tools for RCTs [19], some studies may have been missed; this would not likely change our general findings.

Data extraction and item classification was performed independently by two researchers with disagreements resolved by consensus. The process of classifying items was somewhat subjective; therefore, classification of some items may be debated. Difficulties in classifying items as potentially linked to bias have been acknowledged in previous studies that analyzed bias in different types of research designs [25,28,65].

We used Scopus database to track all original papers describing quality tools. We acknowledge that this approach is only an indirect measure of the usage of quality tools and should not be interpreted as absolute indicator of usage over time.

## Conclusion

There is a considerable number of tools to evaluate the quality of RCTs in health research. There is extensive variation in the number of individual items across quality assessment tools and an apparent lack of agreement between PT and general health research tools in the type of items that are included. There is a need for clarity and consistency of the constructs evaluated by items in quality assessment tools, particularly for aspects related to internal validity, external validity, precision, and quality of reporting. The selection of items to assess internal validity, or risk of bias, should be based on empirical evidence of an association with distortions of treatment effects. Finally, tools and items should undergo a thorough validation process to examine their psychometric properties. Future studies in this area should investigate which items are linked to bias through empirical evidence or psychometric testing. This information will be valuable for the field of knowledge synthesis.

### What is new?

#### Key findings

There is extensive item variation across tools that evaluate the risk of bias of RCTs. There is a lack of empirical evidence to support the association with bias for many items.

What this adds to what is known: Although some studies have previously addressed the use of tools for quality assessment of RCTs, this is the first study that exhaustively explores the type and frequency of items included in different tools that evaluate the risk of bias of RCTs in health research. The number of items included across quality tools is large: 130 and 48 different items have been used by general health research and physical therapy (PT) tools, respectively. Many items are used without a clear identification of their link to bias, or internal validity. The frequency of use of these items varies according to health area (as demonstrated by our comparison between PT and general health research), which suggests a lack of agreement regarding their relevance to trial quality or risk of bias.

What is the implication, what should change now? Results of this study call for an in-depth empirical analysis of the items that should be used to assess risk of bias of RCTs in health research. This information is urgently needed to develop guidelines for the design, conduct, and implementation of trials. In addition, this information is important for systematic reviewers and meta-analysts to evaluate the risk of bias of intervention trials in different areas of health research.

## Abbreviations

CCDAN: Cochrane collaboration depression, anxiety, and neurosis; PT: Physical therapy; RCT: Randomized controlled trial; RCT-NP: Randomized controlled trial -natural products; RCT-PQRS: Randomized controlled trial psychotherapy quality rating scale; RoB: Risk of bias; SRs: Systematic reviews.

## Competing interests

The authors declare that they have no competing interests.

## Authors’ contributions

AO conceived of the study, designed the study, and drafted the manuscript. AO, JF, MO and HS contributed to data collection data analysis, and interpretation. LH provided feedback on the concept and research design and participated in interpretation of data. All authors critically revised the manuscript and provided final approval of the version to be published.

## Author’s information

Susan Armijo-Olivo has a Bsc in Physical therapy (PT) from the Pontifical University Catholic of Chile, a MSc PT and a PhD in Rehabilitation Sciences from the University of Alberta. Her major field of research is diagnosis, evaluation, and treatment of patients with musculoskeletal pain especially temporomandibular disorders and cervical spine disorders along with physical therapy evidence based practice. She currently is a postdoctoral fellow at the CLEAR (Connnecting Leaadership and Research Program in the Faculty of Nursing at the University of Alberta. Her post-doctoral project will focus on the methodological predictors of effect size estimates in PT trials. This research is critical to accurately provide conclusions for health care and decision making. This project will be an important contribution to the area of knowledge synthesis and translation in the PT field and allied health professions.

## Pre-publication history

The pre-publication history for this paper can be accessed here:

http://www.biomedcentral.com/1471-2288/13/116/prepub

## Supplementary Material

Additional file 1 Search Strategy Example. Click here for file

Additional file 2 **Definition of psychometric properties according to Terwee et al., **[24]. Click here for file

Additional file 3 Bias definitions. Click here for file

Additional file 4 Excluded Studies. Click here for file

Additional file 5 Heath Sciences Tools and items to Measure Methodological Quality of RCTs. Click here for file

Additional file 6 Tools and Items to Assess Quality of RCTs in Physical Therapy. Click here for file
