# Supplementary material for: Inconsistency in the items included in tools used in general health research and physical therapy to evaluate the methodological quality of randomized controlled trials: a descriptive analysis
Source: BMC Med Res Methodol. 2013 Sep 17;13:116. doi: 10.1186/1471-2288-13-116 (PMC3848693; doi:10.1186/1471-2288-13-116)
Supplement: Additional file 2 — Definition of psychometric properties according to Terwee et al., [24]. [file 1471-2288-13-116-S2.doc]

**Additional file 2. Definition of psychometric properties according to Terwee et al., [24]**

| **Psychometric property** | **Definition** | **Criteria for scoring the psychometric properties** |
| --- | --- | --- |
| Internal Consistency | “The extent to which items in a (sub)scale are intercorrelated, thus measuring the same construct” | Factor analyses performed on adequate sample size AND Cronbach’s alpha(s) calculated per dimension AND Cronbach’s alpha(s) between 0.70 and 0.95; |
| Content validity | “The extent to which the domain of interest is comprehensively sampled by the items in the questionnaire” | A clear description is provided of the measurement aim, the target population, the concepts that are being measured, the item selection and who were involved in item selection (i.e. investigators OR experts) |
| Criterion validity | “The extent to which scores on a particular questionnaire relate to a gold standard” | Convincing arguments that gold standard is ‘‘gold’’ AND correlation with gold standard >0.70; |
| Construct validity | “The extent to which scores on a particular questionnaire relate to other measures in a manner that is consistent with theoretically derived hypotheses concerning the concepts that are being  measured” | Specific hypotheses were formulated AND at least 75% of the results are in accordance with these hypotheses |
| Reproducibility |  |  |
| a. Agreement | “The extent to which the scores on repeated measures are close to each other (absolute measurement error)” | Convincing arguments that agreement is acceptable. |
| b. Reliability |  |  |
| Test-retest Reliability | “The extent of agreement across two administrations of a test, assuming nothing happened between testings (like treatment or other change-producing event).” | ICC or Kappa >0.70 |
| Inter-rater Reliability | “The extent of agreement among two or more raters at a single testing session.” | ICC or Kappa >0.70 |

ICC = intraclass correlation coefficient
