# Supplementary material for: Inconsistency in the items included in tools used in general health research and physical therapy to evaluate the methodological quality of randomized controlled trials: a descriptive analysis
Source: BMC Med Res Methodol. 2013 Sep 17;13:116. doi: 10.1186/1471-2288-13-116 (PMC3848693; doi:10.1186/1471-2288-13-116)
Supplement: Additional file 3 — Bias definitions. [file 1471-2288-13-116-S3.doc]

Additional file 3. Bias definitions

| Threats to validity ( systematic error) |
| --- |
| Selection Bias |
| “Systematic differences in baseline characteristics of the groups that are compared (for multiple-arm studies) or within the group (for single-arm or cross-sectional studies). For example, from self- selection of treatments, physician-directed selection of treatments, or demographic characteristics, failure to account for intention-to-treat clinical, or social characteristics. Random sequence generation and allocation concealment avoid selection bias.”[10]  “Includes confounding from differential selection before exposure and disease as well as selection bias where exposure and/or disease influence the selection of the participants.” [27] |
| Performance Bias |
| “Performance bias refers to systematic differences between groups in the care that is provided, or in exposure to factors other than the interventions of interest. After enrolment into the study, blinding (or masking) of study participants and personnel may reduce the risk that knowledge of which intervention was received, rather than the intervention itself, affects outcomes. Effective blinding can also ensure that the compared groups receive a similar amount of attention, ancillary treatment and diagnostic investigations.” [10]  “Systematic differences in the care provided to participants in the comparison groups other than the intervention under investigation (for multiple-arm studies) or within groups (for single-arm and cross-sectional studies), e.g., variation in delivery of the protocol, difference in co-interventions, inadequate blinding of providers and participants (variation unlikely in observational studies)” [27] |
| Detection Bias |
| “Systematic differences in outcomes assessment among the comparison groups (for multiple-arm studies) or within groups (for single-arm and cross-sectional studies, e.g., inadequate assessor blinding, differential outcome assessment)”. [27]  “Detection bias refers to systematic differences between groups in how outcomes are determined. Blinding (or masking) of outcome assessors may reduce the risk that knowledge of which intervention was received, rather than the intervention itself, affects outcome measurement. Blinding of outcome assessors can be especially important for assessment of subjective outcomes, such as degree of pain.” [10] |
| Attrition Bias |
| “Attrition bias refers to systematic differences between groups in withdrawals from a study. Withdrawals from the study lead to incomplete outcome data. There are two reasons for withdrawals or incomplete outcome data in clinical trials. Exclusions refer to situations in which some participants are omitted from reports of analyses, despite outcome data being available to the trialists. Attrition refers to situations in which outcome data are not available” [10]  “Systematic differences among the comparison groups in the loss of participants from the study (for multiple-arm studies) or within groups (for single-arm and cross-sectional studies) and how they were accounted for in the results, e.g., incomplete follow-up, differential attrition” [27] |
| Reporting Bias |
| “Reporting bias refers to systematic differences between reported and unreported findings. Within a published report those analyses with statistically significant differences between intervention groups are more likely to be reported than non-significant differences. This sort of ‘within-study publication bias’ is usually known as outcome reporting bias or selective reporting bias, and may be one of the most substantial biases affecting results from individual studies.” [10]  “Systematic differences between reported and unreported findings, e.g., differential reporting of outcomes or harms, potential for bias in reporting through source of funding” [27] |
| Information Bias |
| Systematic differences caused by measurement errors, e.g., recall bias [27] |
| Threats to Precision (random Error) |
| Inadequate study size ( not powered to test study hypothesis) [27] |
