# Supplementary material for: Inconsistency in the items included in tools used in general health research and physical therapy to evaluate the methodological quality of randomized controlled trials: a descriptive analysis
Source: BMC Med Res Methodol. 2013 Sep 17;13:116. doi: 10.1186/1471-2288-13-116 (PMC3848693; doi:10.1186/1471-2288-13-116)
Supplement: Additional file 4 — Excluded Studies. [file 1471-2288-13-116-S4.docx]

# Additional file 4**: Excluded Studies**

**Excluded Research Studies**

The application of the selection criteria resulted in 148 excluded studies. The primary reasons for exclusion of studies were as follows: 1) the study used a quality tool for which information on construction, development and/or psychometric properties was not available (n = 40); 2) the tool was already included in the original review (n = 39); 3) the study used a tool that was not specific for quality assessment of randomized controlled trials (n = 23); 4) the study used modified quality already included (n = 20); 5) the study used an instrument that was not a quality tool (n = 11); 6) the study used a tool developed for single review purposes (n = 8); 7) the study was animal research (n = 4); 8) the study did not focus on a particular tool (n = 2); and 9) information on the name of the scale was not provided (n = 1).

| **Main reason for exclusion:**  **The study used a quality tool for which information on construction, development and/or psychometric properties was not available (n = 40)** |
| --- |
| 1. Korotkov KG, Matravers P, Orlov DV, Williams BO. Application of electrophoton capture (EPC) analysis based on gas discharge visualization (GDV) technique in medicine: a systematic review. J Altern Complement Med. 2010;16(1):13-25 |
| 1. O A, A G, M J, H WC, Al-Kandari A, S C, M M. Is laparoscopic inguinal hernia repair more effective than open repair? J Coll Physicians Surg Pak. 2011;21(5):291-6. |
| 1. Baker A, Potter J, Young K, Madan I. The applicability of grading systems for guidelines. J Eval Clin Pract. 2011;17(4):758-62. |
| 1. Beake S, Pellowe C, Dykes F, Schmied V, Bick D. A systematic review of structured compared with non-structured breastfeeding programmes to support the initiation and duration of exclusive and any breastfeeding in acute and primary health care settings. Matern Child Nutr. 2012;8(2):141-61. |
| 1. Bhagat S, Agarwal M, Roy V. Serratiopeptidase: a systematic review of the existing evidence. Int J Surg. 2013;11(3):209-17. |
| 1. Brantingham JW, Cassa TK, Bonnefin D, Jensen M, Globe G, Hicks M, Korporaal C. Manipulative therapy for shoulder pain and disorders: expansion of a systematic review. J Manipulative Physiol Ther. 2011;34(5):314-46. |
| 1. Carlesso LC, Gross AR, Santaguida PL, Burnie S, Voth S, Sadi J. Adverse events associated with the use of cervical manipulation and mobilization for the treatment of neck pain in adults: a systematic review. Man Ther. 2010;15(5):434-44. |
| 1. Chan MF, Wong ZY, Thayala NV. The effectiveness of music listening in reducing depressive symptoms in adults: a systematic review. Complement Ther Med. 2011;19(6):332-48. |
| 1. Cooper C, Katona C, Lyketsos K, Blazer D, Brodaty H, Rabins P, de Mendonça Lima CA, Livingston G. A systematic review of treatments for refractory depression in older people. Am J Psychiatry. 2011;168(7):681-8. |
| 1. Cooper C, Mukadam N, Katona C, Lyketsos CG, Ames D, Rabins P, Engedal K, de Mendonça Lima C, Blazer D, Teri L, Brodaty H, Livingston G; World Federation of Biological Psychiatry – Old Age Taskforce. Systematic review of the effectiveness of non-pharmacological interventions to improve quality of life of people with dementia. Int Psychogeriatr. 2012;24(6):856-70. |
| 1. Ding M, Leach M, Bradley H. The effectiveness and safety of ginger for pregnancy-induced nausea and vomiting: a systematic review. Women Birth. 2013;26(1):e26-30. |
| 1. Fitzmaurice GJ, McWilliams B, Hurreiz H, Epanomeritakis E. Antibiotics versus appendectomy in the management of acute appendicitis: a review of the current evidence. Can J Surg. 2011;54(5):307-14. |
| 1. Franic M, Kujundzic Tiljak M, Pozar M, Romic D, Mimica M, Petrak J, Ivankovic D, Pecina M. Anterior versus posterior approach in 3D correction of adolescent idiopathic thoracic scoliosis: a meta-analysis. Orthop Traumatol Surg Res. 2012;98(7):795-802. |
| 1. Ghanizadeh A. Hyperbaric oxygen therapy for treatment of children with autism: a systematic review of randomized trials. Med Gas Res. 2012 May 11;2:13. |
| 1. Hajebrahimi S, Mokhtarkhani M, Torabie Z, Taleschian Tabrizi N, Beig Zali S. What is the level of evidence for advertisements in SIU and EAU journals published in 2010 and 2011? preliminary results. SIU World Meeting 2012; Fukoka, Japan; Sept 3-Oct 4, 2012. Abstract 01803. |
| 1. Hall B, Chesters J, Robinson A. Infantile colic: a systematic review of medical and conventional therapies. J Paediatr Child Health. 2012;48(2):128-37. |
| 1. Halton K, Sarna M, Barnett A, Graves N, Leonardo L. Systematic review of community-based strategies to control emerging zoonotic infectious diseases in Southeast Asia. 15^th^ International Congress on Infectious Diseases; Bangkog, Thailand; June 13-16, 2012. Abstract: 46.030. |
| 1. Hawk C, Khorsan R, Lisi AJ, Ferrance RJ, Evans MW. Chiropractic care for nonmusculoskeletal conditions: a systematic review with implications for whole systems research. J Altern Complement Med. 2007;13(5):491-512. |
| 1. Joseph MF, Taft K, Moskwa M, Denegar CR. Deep friction massage to treat tendinopathy: a systematic review of a classic treatment in the face of a new paradigm of understanding. J Sport Rehabil. 2012;21(4):343-53. |
| 1. Kehinde JO, Pope C, Amella EJ. Methodological issues in fall prevention research involving older adults in long-term care facilities. Res Gerontol Nurs. 2011;4(4):294-304 |
| 1. Khorsan R, Coulter ID, Crawford C, Hsiao AF. Systematic review of integrative health care research: randomized control trials, clinical controlled trials, and meta-analysis. Evid Based Complement Alternat Med. 2011;2011. pii: 636134. doi: 10.1155/2011/636134. |
| 1. Krungkraipetch N, Krungkraipetch K, Kaewboonchoo O, Arphorn S, Sim M. Interventions to prevent musculoskeletal disorders among informal sector workers: a literature review. Southeast Asian J Trop Med Public Health. 2012;43(2):510-25. |
| 1. Lapkin S, Levett-Jones T, Gilligan C. A systematic review of the effectiveness of interprofessional education in health professional programs. Nurse Educ Today. 2013;33(2):90-102. |
| 1. [Lee C](http://www.ncbi.nlm.nih.gov/pubmed?term=Lee%20C%5BAuthor%5D&cauthor=true&cauthor_uid=23067573), [Crawford C](http://www.ncbi.nlm.nih.gov/pubmed?term=Crawford%20C%5BAuthor%5D&cauthor=true&cauthor_uid=23067573), [Wallerstedt D](http://www.ncbi.nlm.nih.gov/pubmed?term=Wallerstedt%20D%5BAuthor%5D&cauthor=true&cauthor_uid=23067573), [York A](http://www.ncbi.nlm.nih.gov/pubmed?term=York%20A%5BAuthor%5D&cauthor=true&cauthor_uid=23067573), [Duncan A](http://www.ncbi.nlm.nih.gov/pubmed?term=Duncan%20A%5BAuthor%5D&cauthor=true&cauthor_uid=23067573), [Smith J](http://www.ncbi.nlm.nih.gov/pubmed?term=Smith%20J%5BAuthor%5D&cauthor=true&cauthor_uid=23067573), [Sprengel M](http://www.ncbi.nlm.nih.gov/pubmed?term=Sprengel%20M%5BAuthor%5D&cauthor=true&cauthor_uid=23067573), [Welton R](http://www.ncbi.nlm.nih.gov/pubmed?term=Welton%20R%5BAuthor%5D&cauthor=true&cauthor_uid=23067573), [Jonas W](http://www.ncbi.nlm.nih.gov/pubmed?term=Jonas%20W%5BAuthor%5D&cauthor=true&cauthor_uid=23067573). The effectiveness of acupuncture research across components of the trauma spectrum response (tsr): a systematic review of reviews. Syst Rev. 2012 Oct 15;1:46. doi: 10.1186/2046-4053-1-46. |
| 1. Li YL, Nie YL, Huang Y, Zhang MM, Li J. Patients for patient safety: A systematic review. CJEBM 2011;8:903-9. |
| 1. [Markides GA](http://www.ncbi.nlm.nih.gov/pubmed?term=Markides%20GA%5BAuthor%5D&cauthor=true&cauthor_uid=21407246), [Alkhaffaf B](http://www.ncbi.nlm.nih.gov/pubmed?term=Alkhaffaf%20B%5BAuthor%5D&cauthor=true&cauthor_uid=21407246), [Vickers J](http://www.ncbi.nlm.nih.gov/pubmed?term=Vickers%20J%5BAuthor%5D&cauthor=true&cauthor_uid=21407246). Nutritional access routes following oesophagectomy--a systematic review. Eur J Clin Nutr. 201;65(5):565-73. |
| 1. McClellan R. Exercise programs for patients with cancer improve physical functioning and quality of life. J Physiother. 2013;59(1):57. |
| 1. O A, A G, M J, H WC, Al-Kandari A, S C, M M. Is laparoscopic inguinal hernia repair more effective than open repair? J Coll Physicians Surg Pak. 2011;21(5):291-6. |
| 1. Parekh S. The effect of acupuncture (vs. different types of controls) on knee pain-a literature review. Can evidence-based medicine help us to understand the mechanism of acupuncture better? Evidence Live 2011. Oxford, UK. Mar 25-26, 2011. |
| 1. Pelton T, van Vliet P, Hollands K. Interventions for improving coordination of reach to grasp following stroke: a systematic review. Int J Evid Based Healthc. 2012;10(2):89-102. |
| 1. [Pringsheim T](http://www.ncbi.nlm.nih.gov/pubmed?term=Pringsheim%20T%5BAuthor%5D&cauthor=true&cauthor_uid=21751826), [Lam D](http://www.ncbi.nlm.nih.gov/pubmed?term=Lam%20D%5BAuthor%5D&cauthor=true&cauthor_uid=21751826), [Ching H](http://www.ncbi.nlm.nih.gov/pubmed?term=Ching%20H%5BAuthor%5D&cauthor=true&cauthor_uid=21751826), [Patten S](http://www.ncbi.nlm.nih.gov/pubmed?term=Patten%20S%5BAuthor%5D&cauthor=true&cauthor_uid=21751826). Metabolic and neurological complications of second-generation antipsychotic use in children: a systematic review and meta-analysis of randomized controlled trials. Drug Saf. 2011;34(8):651-68 |
| 1. Richards E, van Kessel G, Virgara R, Harris P. Does antenatal physical therapy for pregnant women with low back pain or pelvic pain improve functional outcomes? A systematic review. Acta Obstet Gynecol Scand. 2012;91(9):1038-45. |
| 1. Robinson N, Lorenc A, Liao X. The evidence for Shiatsu: a systematic review of Shiatsu and acupressure. BMC Complement Altern Med. 2011 Oct 7;11:88. doi: 10.1186/1472-6882-11-88. |
| 1. Rothenberger LG, Henschel AD, Schrey D, Becker A, Boos J. Methodological and ethical aspects of randomized controlled clinical trials in minors with malignant diseases. Pediatr Blood Cancer. 2011;57(4):599-605. |
| 1. Sameem M, Wood T, Ignacy T, Thoma A, Strumas N. A systematic review of rehabilitation protocols after surgical repair of the extensor tendons in zones V-VIII of the hand. J Hand Ther. 2011;24(4):365-72 |
| 1. Smith TO, Taylor R, Hing CB. Thromboprophylaxis following major skeletal trauma: a systematic review. Eur J Trauma Emerg Surg 2011;37(5):479-90. |
| 1. Sultana SS, MacDermid JC, Grewal R, Rath S. The effectiveness of early mobilization after tendon transfers in the hand: a systematic review. J Hand Ther. 2013;26(1):1-20 |
| 1. Wong S, Ordean A, Kahan M. Substance use in pregnancy, J Obstet Gynaecol Can 2011 Apr;33(4):367-84 |
| 1. Yoon U, Kwok LL, Magkidis A. Efficacy of lifestyle interventions in reducing diabetes incidence in patients with impaired glucose tolerance: A systematic review of randomized controlled trials. BMC Proceedings 2012, 6(Suppl 3):P28 doi:10.1186/1753-6561-6-S3-P28 |
| 1. Yu RX, Muller-Riemenschneider F.  Effectiveness of exercise after PCI in the secondary prevention of coronary heart disease: a systematic review. Eur J Integ Med 2011; 3(2): e63-9. |

| **Main reason for exclusion:**  **The tool was already included in the original review (n = 39)** |
| --- |
| 1. Balfour-Lynn IM, Welch K. Inhaled corticosteroids for cystic fibrosis. Cochrane Database Syst Rev. 2012 Nov 14;11:CD001915. |
| 1. Beale M, Cella M, Williams AC. Comparing patients' and clinician-researchers' outcome choice for psychological treatment of chronic pain. Pain. 2011;152(10):2283-6. |
| 1. Bellemain-Appaix A, O'Connor SA, Silvain J, Cucherat M, Beygui F, Barthélémy O, Collet JP, Jacq L, Bernasconi F, Montalescot G; ACTION Group. Association of clopidogrel pretreatment with mortality, cardiovascular events, and major bleeding among patients undergoing percutaneous coronary intervention: A systematic review and meta-analysis. JAMA. 2012;308(23):2507-16 |
| 1. Booth S, Simon S, Higginson IJ, Harding R, Bausewein C. The efficacy of benzodiazepines for palliating dyspnoea: A systematic review. Eur J Cancer 2011;47(Suppl1): S77. |
| 1. Chan CMK, Yu SC, LeeA. A systematic review of the analgesic efficacy and adverse effects of epidural morphine versus parenteral morphine after caesarean section. Anaes Intensive Care 2011;39(4). |
| 1. Derosa G, Maffioli P. Efficacy and safety profile evaluation of acarbose alone and in association with other antidiabetic drugs: a systematic review. Clin Ther. 2012;34(6):1221-36 |
| 1. Diener MK, Heukaufer C, Schwarzer G, Seiler CM, Antes G, Buchler M, Knaebel HP. Pancreaticoduodenectomy (classic Whipple) versus pylorus-preserving pancreaticoduodenectomy (pp Whipple) for surgical treatment of periampullary and pancreatic carcinoma. Cochrane Database Syst Rev. 2008;(2):CD006053. |
| 1. Fachini A, Aliane PP, Martinez EZ, Furtado EF. Efficacy of brief alcohol screening intervention for college students (BASICS): a meta-analysis of randomized controlled trials. Subst Abuse Treat Prev Policy. 2012;7:40. |
| 1. [Geraghty AJ](http://www.ncbi.nlm.nih.gov/pubmed?term=Geraghty%20AJ%5BAuthor%5D&cauthor=true&cauthor_uid=21678330), [Welch K](http://www.ncbi.nlm.nih.gov/pubmed?term=Welch%20K%5BAuthor%5D&cauthor=true&cauthor_uid=21678330). Antithrombotic agents for preventing thrombosis after infrainguinal arterial bypass surgery. Cochrane Database Syst Rev. 2011 ;(6):CD000536. |
| 1. [Giometto B](http://www.ncbi.nlm.nih.gov/pubmed?term=Giometto%20B%5BAuthor%5D&cauthor=true&cauthor_uid=23235647), [Vitaliani R](http://www.ncbi.nlm.nih.gov/pubmed?term=Vitaliani%20R%5BAuthor%5D&cauthor=true&cauthor_uid=23235647), [Lindeck-Pozza E](http://www.ncbi.nlm.nih.gov/pubmed?term=Lindeck-Pozza%20E%5BAuthor%5D&cauthor=true&cauthor_uid=23235647), [Grisold W](http://www.ncbi.nlm.nih.gov/pubmed?term=Grisold%20W%5BAuthor%5D&cauthor=true&cauthor_uid=23235647), [Vedeler C](http://www.ncbi.nlm.nih.gov/pubmed?term=Vedeler%20C%5BAuthor%5D&cauthor=true&cauthor_uid=23235647). Treatment for paraneoplastic neuropathies. Cochrane Database Syst Rev. 2012;12:CD007625 |
| 1. [Gonçalves R](http://www.ncbi.nlm.nih.gov/pubmed?term=Gon%C3%A7alves%20R%5BAuthor%5D&cauthor=true&cauthor_uid=23300515), [Pedrozo AL](http://www.ncbi.nlm.nih.gov/pubmed?term=Pedrozo%20AL%5BAuthor%5D&cauthor=true&cauthor_uid=23300515), [Coutinho ES](http://www.ncbi.nlm.nih.gov/pubmed?term=Coutinho%20ES%5BAuthor%5D&cauthor=true&cauthor_uid=23300515), [Figueira I](http://www.ncbi.nlm.nih.gov/pubmed?term=Figueira%20I%5BAuthor%5D&cauthor=true&cauthor_uid=23300515), [Ventura P](http://www.ncbi.nlm.nih.gov/pubmed?term=Ventura%20P%5BAuthor%5D&cauthor=true&cauthor_uid=23300515). Efficacy of virtual reality exposure therapy in the treatment of PTSD: a systematic review. PLoS One. 2012;7(12):e48469. |
| 1. Gougoulias N, Khanna A, McBride DJ, Maffulli N. Management of calcaneal fractures: systematic review of randomized trials. Br Med Bull. 2009;92:153-67. |
| 1. [Greenfield ML](http://www.ncbi.nlm.nih.gov/pubmed?term=Greenfield%20ML%5BAuthor%5D&cauthor=true&cauthor_uid=19448222), [Mhyre JM](http://www.ncbi.nlm.nih.gov/pubmed?term=Mhyre%20JM%5BAuthor%5D&cauthor=true&cauthor_uid=19448222), [Mashour GA](http://www.ncbi.nlm.nih.gov/pubmed?term=Mashour%20GA%5BAuthor%5D&cauthor=true&cauthor_uid=19448222), [Blum JM](http://www.ncbi.nlm.nih.gov/pubmed?term=Blum%20JM%5BAuthor%5D&cauthor=true&cauthor_uid=19448222), [Yen EC](http://www.ncbi.nlm.nih.gov/pubmed?term=Yen%20EC%5BAuthor%5D&cauthor=true&cauthor_uid=19448222), [Rosenberg AL](http://www.ncbi.nlm.nih.gov/pubmed?term=Rosenberg%20AL%5BAuthor%5D&cauthor=true&cauthor_uid=19448222). Improvement in the quality of randomized controlled trials among general anesthesiology journals 2000 to 2006: a 6-year follow-up. Anesth Analg. 2009;108(6):1916-21. |
| 1. [Guo X](http://www.ncbi.nlm.nih.gov/pubmed?term=Guo%20X%5BAuthor%5D&cauthor=true&cauthor_uid=18199012), [Zhou B](http://www.ncbi.nlm.nih.gov/pubmed?term=Zhou%20B%5BAuthor%5D&cauthor=true&cauthor_uid=18199012), [Nishimura T](http://www.ncbi.nlm.nih.gov/pubmed?term=Nishimura%20T%5BAuthor%5D&cauthor=true&cauthor_uid=18199012), [Teramukai S](http://www.ncbi.nlm.nih.gov/pubmed?term=Teramukai%20S%5BAuthor%5D&cauthor=true&cauthor_uid=18199012), [Fukushima M](http://www.ncbi.nlm.nih.gov/pubmed?term=Fukushima%20M%5BAuthor%5D&cauthor=true&cauthor_uid=18199012). Clinical effect of qigong practice on essential hypertension: a meta-analysis of randomized controlled trials. J Altern Complement Med. 2008;14(1):27-37. |
| 1. [Haidich AB](http://www.ncbi.nlm.nih.gov/pubmed?term=Haidich%20AB%5BAuthor%5D&cauthor=true&cauthor_uid=21172601), [Birtsou C](http://www.ncbi.nlm.nih.gov/pubmed?term=Birtsou%20C%5BAuthor%5D&cauthor=true&cauthor_uid=21172601), [Dardavessis T](http://www.ncbi.nlm.nih.gov/pubmed?term=Dardavessis%20T%5BAuthor%5D&cauthor=true&cauthor_uid=21172601), [Tirodimos I](http://www.ncbi.nlm.nih.gov/pubmed?term=Tirodimos%20I%5BAuthor%5D&cauthor=true&cauthor_uid=21172601), [Arvanitidou M](http://www.ncbi.nlm.nih.gov/pubmed?term=Arvanitidou%20M%5BAuthor%5D&cauthor=true&cauthor_uid=21172601). The quality of safety reporting in trials is still suboptimal: Survey of major general medical journals. J Clin Epidemiol. 2011;64(2):124-35. |
| 1. [Hammerschlag R](http://www.ncbi.nlm.nih.gov/pubmed?term=Hammerschlag%20R%5BAuthor%5D&cauthor=true&cauthor_uid=20953418), [Milley R](http://www.ncbi.nlm.nih.gov/pubmed?term=Milley%20R%5BAuthor%5D&cauthor=true&cauthor_uid=20953418), [Colbert A](http://www.ncbi.nlm.nih.gov/pubmed?term=Colbert%20A%5BAuthor%5D&cauthor=true&cauthor_uid=20953418), [Weih J](http://www.ncbi.nlm.nih.gov/pubmed?term=Weih%20J%5BAuthor%5D&cauthor=true&cauthor_uid=20953418), [Yohalem-Ilsley B](http://www.ncbi.nlm.nih.gov/pubmed?term=Yohalem-Ilsley%20B%5BAuthor%5D&cauthor=true&cauthor_uid=20953418), [Mist S](http://www.ncbi.nlm.nih.gov/pubmed?term=Mist%20S%5BAuthor%5D&cauthor=true&cauthor_uid=20953418), [Aickin M](http://www.ncbi.nlm.nih.gov/pubmed?term=Aickin%20M%5BAuthor%5D&cauthor=true&cauthor_uid=20953418). Randomized Controlled Trials of Acupuncture (1997-2007): An Assessment of Reporting Quality with a CONSORT- and STRICTA-Based Instrument. Evid Based Complement Alternat Med. 2011;2011. pii: 183910. doi: 10.1155/2011/183910. |
| 1. [Harding R](http://www.ncbi.nlm.nih.gov/pubmed?term=Harding%20R%5BAuthor%5D&cauthor=true&cauthor_uid=21737481), [List S](http://www.ncbi.nlm.nih.gov/pubmed?term=List%20S%5BAuthor%5D&cauthor=true&cauthor_uid=21737481), [Epiphaniou E](http://www.ncbi.nlm.nih.gov/pubmed?term=Epiphaniou%20E%5BAuthor%5D&cauthor=true&cauthor_uid=21737481), [Jones H](http://www.ncbi.nlm.nih.gov/pubmed?term=Jones%20H%5BAuthor%5D&cauthor=true&cauthor_uid=21737481). How can informal caregivers in cancer and palliative care be supported? An updated systematic literature review of interventions and their effectiveness. Palliat Med. 2012;26(1):7-22. |
| 1. [Harris B](http://www.ncbi.nlm.nih.gov/pubmed?term=Harris%20B%5BAuthor%5D&cauthor=true&cauthor_uid=23171713), [Andrews PJ](http://www.ncbi.nlm.nih.gov/pubmed?term=Andrews%20PJ%5BAuthor%5D&cauthor=true&cauthor_uid=23171713), [Murray GD](http://www.ncbi.nlm.nih.gov/pubmed?term=Murray%20GD%5BAuthor%5D&cauthor=true&cauthor_uid=23171713), [Forbes J](http://www.ncbi.nlm.nih.gov/pubmed?term=Forbes%20J%5BAuthor%5D&cauthor=true&cauthor_uid=23171713), [Moseley O](http://www.ncbi.nlm.nih.gov/pubmed?term=Moseley%20O%5BAuthor%5D&cauthor=true&cauthor_uid=23171713). Systematic review of head cooling in adults after traumatic brain injury and stroke. Health Technol Assess. 2012;16(45):1-175 |
| 1. Hart MG, Grant R, Garside R, Rogers G, Somerville M, Stein K. Chemotherapy wafers for high grade glioma. Cochrane Database Syst Rev. 2011;(3):CD007294 |
| 1. Hart MG, Grant R, Metcalfe ES. Biopsy versus resection for high grade glioma. Cochrane Database Syst Rev 2009;(2):CD002034. |
| 1. Hart MG, Grant R, Walker M, Dickinson H. Surgical resection and whole brain radiation therapy versus whole brain radiation therapy alone for single brain metastases. Cochrane Database Syst Rev. 2005;(1):CD003292. |
| 1. Helm S, Hayek SM, Colson J, Chopra P, Deer TR, Justiz R, Hameed M, Falco FJ. Spinal endoscopic adhesiolysis in post lumbar surgery syndrome: an update of assessment of the evidence. Pain Physician. 2013;16(2 Suppl):SE125-50. |
| 1. [Herbert RD](http://www.ncbi.nlm.nih.gov/pubmed?term=Herbert%20RD%5BAuthor%5D&cauthor=true&cauthor_uid=21735398), [de Noronha M](http://www.ncbi.nlm.nih.gov/pubmed?term=de%20Noronha%20M%5BAuthor%5D&cauthor=true&cauthor_uid=21735398), [Kamper SJ](http://www.ncbi.nlm.nih.gov/pubmed?term=Kamper%20SJ%5BAuthor%5D&cauthor=true&cauthor_uid=21735398). Stretching to prevent or reduce muscle soreness after exercise. Cochrane Database Syst Rev. 2011;(7):CD004577. |
| 1. [Hoogeboom TJ](http://www.ncbi.nlm.nih.gov/pubmed?term=Hoogeboom%20TJ%5BAuthor%5D&cauthor=true&cauthor_uid=22675429), [Oosting E](http://www.ncbi.nlm.nih.gov/pubmed?term=Oosting%20E%5BAuthor%5D&cauthor=true&cauthor_uid=22675429), [Vriezekolk JE](http://www.ncbi.nlm.nih.gov/pubmed?term=Vriezekolk%20JE%5BAuthor%5D&cauthor=true&cauthor_uid=22675429), [Veenhof C](http://www.ncbi.nlm.nih.gov/pubmed?term=Veenhof%20C%5BAuthor%5D&cauthor=true&cauthor_uid=22675429), [Siemonsma PC](http://www.ncbi.nlm.nih.gov/pubmed?term=Siemonsma%20PC%5BAuthor%5D&cauthor=true&cauthor_uid=22675429), [de Bie RA](http://www.ncbi.nlm.nih.gov/pubmed?term=de%20Bie%20RA%5BAuthor%5D&cauthor=true&cauthor_uid=22675429), [van den Ende CH](http://www.ncbi.nlm.nih.gov/pubmed?term=van%20den%20Ende%20CH%5BAuthor%5D&cauthor=true&cauthor_uid=22675429), [van Meeteren NL](http://www.ncbi.nlm.nih.gov/pubmed?term=van%20Meeteren%20NL%5BAuthor%5D&cauthor=true&cauthor_uid=22675429). Therapeutic validity and effectiveness of preoperative exercise on functional recovery after joint replacement: a systematic review and meta-analysis. PLoS One. 2012;7(5):e38031. doi: 10.1371/journal.pone.0038031. |
| 1. Howe TE, Shea B, Dawson LJ, Downie F, Murray A, Ross C, Harbour RT, Caldwell LM, Creed G. Exercise for preventing and treating osteoporosis in postmenopausal women. Cochrane Database Syst Rev. 2011;(7):CD000333. |
| 1. Hueskes BA, Roovers EA, Mantel-Teeuwisse AK, Janssens HJ, van de Lisdonk EH, Janssen M. Use of diuretics and the risk of gouty arthritis: a systematic review. Semin Arthritis Rheum. 2012;41(6):879-89. |
| 1. Hui D, Arthur J, Dalal S, Bruera E. Quality of the supportive and palliative oncology literature: a focused analysis on randomized controlled trials. Support Care Cancer. 2012;20(8):1779-85 |
| 1. Ibrahim H, Sinha IP, Subhedar NV. Corticosteroids for treating hypotension in preterm infants. Cochrane Database Syst Rev. 2011;(12):CD003662. |
| 1. Kirchin V, Page T, Keegan PE, Atiemo K, Cody JD, McClinton S. Urethral injection therapy for urinary incontinence in women. Cochrane Database Syst Rev. 2012;2:CD003881. |
| 1. LaRocca R, Yost J, Dobbins M, Ciliska D, Butt M. The effectiveness of knowledge translation strategies used in public health: a systematic review. BMC Public Health. 2012;12:751. |
| 1. Li L, Sun T, Zhang P, Tian J, Yang K. Statins for primary prevention of venous thromboembolism. Cochrane Database Syst Rev. 2011;(12):CD008203. |
| 1. Martin WJ, Forouzanfar T. The efficacy of anticonvulsants on orofacial pain: a systematic review. Oral Surg Oral Med Oral Pathol Oral Radiol Endod. 2011;111(5):627-33. |
| 1. Martin WJ, Perez RS, Tuinzing DB, Forouzanfar T. Efficacy of antidepressants on orofacial pain: a systematic review. Int J Oral Maxillofac Surg. 2012;41(12):1532-9. |
| 1. Minozzi S, Amato L, Vecchi S, Davoli M, Kirchmayer U, Verster A. Oral naltrexone maintenance treatment for opioid dependence. Cochrane Database Syst Rev. 2011;(4):CD001333. |
| 1. Oomens MA, Forouzanfar T. Antibiotic prophylaxis in third molar surgery: a review. Oral Surg Oral Med Oral Pathol Oral Radiol. 2012;114(6):e5-12. |
| 1. Pinto D, Robertson MC, Hansen P, Abbott JH. Cost-effectiveness of nonpharmacologic, nonsurgical interventions for hip and/or knee osteoarthritis: systematic review. Value Health. 2012;15(1):1-12. |
| 1. Soo C, Tate RL, Lane-Brown A. A systematic review of acceptance and commitment therapy (ACT) for managing anxiety: Applicability for people with acquired brain injury? Brain Impairment 2011;12(1)54-70. |
| 1. van Oldenrijk J, van Berkel Y, Kerkhoffs GM, Bhandari M, Poolman RW. Do authors report surgical expertise in open spine surgery related randomized controlled trials? a systematic review on quality of reporting. Spine (Phila Pa 1976). 2013;38(10):857-64. |
| 1. Yan S, Xu D, Sun B. Combination of radiofrequency ablation with transarterial chemoembolization for hepatocellular carcinoma: A meta-analysis. Dig Dis Sci. 2013;58(7):2107-13. |

| **Main reason for exclusion:**  **The study used a tool that was not specific for quality assessment of randomized controlled trials (n = 23)** |
| --- |
| 1. Babatunde OO, Forsyth JJ. Quantitative Ultrasound and bone's response to exercise: a meta analysis. Bone. 2013;53(1):311-8. |
| 1. Baral SD, Wirtz A, Sifakis F, Johns B, Walker D, Beyrer C. The highest attainable standard of evidence (HASTE) for HIV/AIDS interventions: toward a public health approach to defining evidence. Public Health Rep. 2012;127(6):572-84. |
| 1. Bereza BG, Machado M, Einarson TR. Assessing the reporting and scientific quality of meta-analyses of randomized controlled trials of treatments for anxiety disorders. Ann Pharmacother. 2008;42(10):1402-9 |
| 1. Chillón P, Evenson KR, Vaughn A, Ward DS. A systematic review of interventions for promoting active transportation to school. Int J Behav Nutr Phys Act. 2011;8:10. doi: 10.1186/1479-5868-8-10. |
| 1. Craane B, Dijkstra PU, Stappaerts K, De Laat A. Methodological quality of a systematic review on physical therapy for temporomandibular disorders: influence of hand search and quality scales. Clin Oral Investig. 2012;16(1):295-303. |
| 1. Desmeules F, Roy JS, MacDermid JC, Champagne F, Hinse O, Woodhouse LJ. Advanced practice physiotherapy in patients with musculoskeletal disorders: a systematic review. BMC Musculoskelet Disord. 2012;13:107. doi: 10.1186/1471-2474-13-107. |
| 1. DiBardino D, Cohen ER, Didwania A. Meta-analysis: multidisciplinary fall prevention strategies in the acute care inpatient population. J Hosp Med. 2012;7(6):497-503. |
| 1. [Dolan GP](http://www.ncbi.nlm.nih.gov/pubmed?term=Dolan%20GP%5BAuthor%5D&cauthor=true&cauthor_uid=22840895), [Harris RC](http://www.ncbi.nlm.nih.gov/pubmed?term=Harris%20RC%5BAuthor%5D&cauthor=true&cauthor_uid=22840895), [Clarkson M](http://www.ncbi.nlm.nih.gov/pubmed?term=Clarkson%20M%5BAuthor%5D&cauthor=true&cauthor_uid=22840895), [Sokal R](http://www.ncbi.nlm.nih.gov/pubmed?term=Sokal%20R%5BAuthor%5D&cauthor=true&cauthor_uid=22840895), [Morgan G](http://www.ncbi.nlm.nih.gov/pubmed?term=Morgan%20G%5BAuthor%5D&cauthor=true&cauthor_uid=22840895), [Mukaigawara M](http://www.ncbi.nlm.nih.gov/pubmed?term=Mukaigawara%20M%5BAuthor%5D&cauthor=true&cauthor_uid=22840895), [Horiuchi H](http://www.ncbi.nlm.nih.gov/pubmed?term=Horiuchi%20H%5BAuthor%5D&cauthor=true&cauthor_uid=22840895), [Hale R](http://www.ncbi.nlm.nih.gov/pubmed?term=Hale%20R%5BAuthor%5D&cauthor=true&cauthor_uid=22840895), [Stormont L](http://www.ncbi.nlm.nih.gov/pubmed?term=Stormont%20L%5BAuthor%5D&cauthor=true&cauthor_uid=22840895), [Béchard-Evans L](http://www.ncbi.nlm.nih.gov/pubmed?term=B%C3%A9chard-Evans%20L%5BAuthor%5D&cauthor=true&cauthor_uid=22840895), [Chao YS](http://www.ncbi.nlm.nih.gov/pubmed?term=Chao%20YS%5BAuthor%5D&cauthor=true&cauthor_uid=22840895), [Eremin S](http://www.ncbi.nlm.nih.gov/pubmed?term=Eremin%20S%5BAuthor%5D&cauthor=true&cauthor_uid=22840895), [Martins S](http://www.ncbi.nlm.nih.gov/pubmed?term=Martins%20S%5BAuthor%5D&cauthor=true&cauthor_uid=22840895), [Tam JS](http://www.ncbi.nlm.nih.gov/pubmed?term=Tam%20JS%5BAuthor%5D&cauthor=true&cauthor_uid=22840895), [Peñalver J](http://www.ncbi.nlm.nih.gov/pubmed?term=Pe%C3%B1alver%20J%5BAuthor%5D&cauthor=true&cauthor_uid=22840895), [Zanuzdana A](http://www.ncbi.nlm.nih.gov/pubmed?term=Zanuzdana%20A%5BAuthor%5D&cauthor=true&cauthor_uid=22840895), [Nguyen-Van-Tam JS](http://www.ncbi.nlm.nih.gov/pubmed?term=Nguyen-Van-Tam%20JS%5BAuthor%5D&cauthor=true&cauthor_uid=22840895). Vaccination of health care workers to protect patients at increased risk for acute respiratory disease. Emerg Infect Dis. 2012;18(8):1225-34. |
| 1. Evans CD, Watson E, Eurich DT, Taylor JG, Yakiwchuk EM, Shevchuk YM, Remillard A, Blackburn D. Diabetes and cardiovascular disease interventions by community pharmacists: a systematic review. Ann Pharmacother. 2011;45(5):615-28. |
| 1. Gomes M, Grieve R, Nixon R, Edmunds WJ. Statistical methods for cost-effectiveness analyses that use data from cluster randomized trials: a systematic review and checklist for critical appraisal. Med Decis Making. 2012;32(1):209-20. |
| 1. Hewitt K, Binns C, Scott J, Forbes D. Pacifier use, breastfeeding and sudden infant death syndrome. Curr Pediatr Rev 2012;8(4):285-91. |
| 1. Howell M, Howard K, Wong G, Turner R, Craig J, Tong A. Quality of life outcomes reported in randomized controlled trials of immunosuppressive drug regimens in kidney transplantation. Transplantation 2012;94(10S): 182. |
| 1. Hoy D, Brooks P, Woolf A, Blyth F, March L, Bain C, Baker P, Smith E, Buchbinder R. Assessing risk of bias in prevalence studies: modification of an existing tool and evidence of interrater agreement. J Clin Epidemiol. 2012;65(9):934-9 |
| 1. Kerr S, Lawrence M, Darbyshire C, Middleton AR, Fitzsimmons L. Tobacco and alcohol-related interventions for people with mild/moderate intellectual disabilities: A systematic review of the literature. J Intellect Disabil Res. 2013;57(5):393-408 |
| 1. Lorenc T, Marrero-Guillamón I, Aggleton P, Cooper C, Llewellyn A, Lehmann A, Lindsay C. Promoting the uptake of HIV testing among men who have sex with men: systematic review of effectiveness and cost-effectiveness. Sex Transm Infect. 2011;87(4):272-8. |
| 1. Louw A, Diener I, Butler DS, Puentedura EJ. The effect of neuroscience education on pain, disability, anxiety, and stress in chronic musculoskeletal pain. Arch Phys Med Rehabil. 2011;92(12):2041-56 |
| 1. Pandor A, Goodacre S, Harnan S, Holmes M, Pickering A, Fitzgerald P, Rees A, Stevenson M. Diagnostic management strategies for adults and children with minor head injury: a systematic review and an economic evaluation. Health Technol Assess. 2011;15(27):1-202 |
| 1. [Sirriyeh R](http://www.ncbi.nlm.nih.gov/pubmed?term=Sirriyeh%20R%5BAuthor%5D&cauthor=true&cauthor_uid=21410846), [Lawton R](http://www.ncbi.nlm.nih.gov/pubmed?term=Lawton%20R%5BAuthor%5D&cauthor=true&cauthor_uid=21410846), [Gardner P](http://www.ncbi.nlm.nih.gov/pubmed?term=Gardner%20P%5BAuthor%5D&cauthor=true&cauthor_uid=21410846), [Armitage G](http://www.ncbi.nlm.nih.gov/pubmed?term=Armitage%20G%5BAuthor%5D&cauthor=true&cauthor_uid=21410846). Reviewing studies with diverse designs: the development and evaluation of a new tool. J Eval Clin Pract. 2012;18(4):746-52. |
| 1. [Sung-Chan P](http://www.ncbi.nlm.nih.gov/pubmed?term=Sung-Chan%20P%5BAuthor%5D&cauthor=true&cauthor_uid=23136914), [Sung YW](http://www.ncbi.nlm.nih.gov/pubmed?term=Sung%20YW%5BAuthor%5D&cauthor=true&cauthor_uid=23136914), [Zhao X](http://www.ncbi.nlm.nih.gov/pubmed?term=Zhao%20X%5BAuthor%5D&cauthor=true&cauthor_uid=23136914), [Brownson RC](http://www.ncbi.nlm.nih.gov/pubmed?term=Brownson%20RC%5BAuthor%5D&cauthor=true&cauthor_uid=23136914). Family-based models for childhood-obesity intervention: a systematic review of randomized controlled trials. Obes Rev. 2012 Nov 9. doi: 10.1111/obr.12000. |
| 1. Testa L, Barbosa CC, Chaves AC, Hoff PM, Riechelmann RP. Health-related quality-of-life (HRQoL) reporting by randomized cancer trials (RCT): Pitfalls for interpretation. J Clin Oncol 29: 2011 (suppl; abstr 6102) |
| 1. Tzelepis F, Paul CL, Walsh RA, McElduff P, Knight J. Proactive telephone counseling for smoking cessation: meta-analyses by recruitment channel and methodological quality. J Natl Cancer Inst. 2011;103(12):922-41. |
| 1. Uchida M, Pogorzelska-Maziarz M, Smith PW, Larson E. Infection prevention in long-term care: a systematic review of randomized and nonrandomized trials. J Am Geriatr Soc. 2013;61(4):602-14. |
| 1. [Wilks DC](http://www.ncbi.nlm.nih.gov/pubmed?term=Wilks%20DC%5BAuthor%5D&cauthor=true&cauthor_uid=21255448), [Mander AP](http://www.ncbi.nlm.nih.gov/pubmed?term=Mander%20AP%5BAuthor%5D&cauthor=true&cauthor_uid=21255448), [Jebb SA](http://www.ncbi.nlm.nih.gov/pubmed?term=Jebb%20SA%5BAuthor%5D&cauthor=true&cauthor_uid=21255448), [Thompson SG](http://www.ncbi.nlm.nih.gov/pubmed?term=Thompson%20SG%5BAuthor%5D&cauthor=true&cauthor_uid=21255448), [Sharp SJ](http://www.ncbi.nlm.nih.gov/pubmed?term=Sharp%20SJ%5BAuthor%5D&cauthor=true&cauthor_uid=21255448), [Turner RM](http://www.ncbi.nlm.nih.gov/pubmed?term=Turner%20RM%5BAuthor%5D&cauthor=true&cauthor_uid=21255448), [Lindroos AK](http://www.ncbi.nlm.nih.gov/pubmed?term=Lindroos%20AK%5BAuthor%5D&cauthor=true&cauthor_uid=21255448). Dietary energy density and adiposity: Employing bias adjustments in a meta-analysis of prospective studies. BMC Public Health. 2011 Jan 22;11:48. doi: 10.1186/1471-2458-11-48. |

| **Main reason for exclusion:**  **The study used modified quality already included (n = 20)** |
| --- |
| Brunoni AR, Tadini L, Fregni F. Changes in clinical trials methodology over time: a systematic review of six decades of research in psychopharmacology. PLoS One. 2010;5(3):e9479. |
| Fu DL, Lu L, Zhu W, Li JH, Li HQ, Liu AJ, Xie C, Zheng GQ. Xiaoxuming decoction for acute ischemic stroke: A systematic review and meta-analysis. J Ethnopharmacol. 2013;148(1):1-13 |
| Hirji KF, Premji ZG. Pre-referral rectal artesunate in severe malaria: flawed trial. Trials. 2011;12:188 |
| Jacobs W, Van der Gaag NA, Tuschel A, de Kleuver M, Peul W, Verbout AJ, Oner FC. Total disc replacement for chronic back pain in the presence of disc degeneration. Cochrane Database Syst Rev. 2012 Sep 12;9:CD008326 |
| Jeste DV, Dunn LB, Folsom DP, Zisook D. Multimedia educational aids for improving consumer knowledge about illness management and treatment decisions: a review of randomized controlled trials. J Psychiatr Res 2008;42(1):1-21. |
| Lee CS, Hwang CJ, Lee DH, Kim YT, Lee HS. Fusion rates of instrumented lumbar spinal arthrodesis according to surgical approach: a systematic review of randomized trials. Clin Orthop Surg. 2011;3(1):39-47 |
| Lee JH, Choi TY, Lee MS, Lee H, Shin BC, Lee H. Acupuncture for acute low back pain: a systematic review. Clin J Pain. 2013;29(2):172-85. |
| Littlewood C, Ashton J, Chance-Larsen K, May S, Sturrock B. Exercise for rotator cuff tendinopathy: a systematic review. Physiotherapy. 2012;98(2):101-9 |
| Margolin A, Avants SK, Kleber HD. Investigating alternative medicine therapies in randomized controlled trials. JAMA 1998;280(18):1626-8. |
| Meeks TW, Wetherell JL, Irwin MR, Redwine LS, Jeste DV. Complementary and alternative treatments for late-life depression, anxiety, and sleep disturbance: a review of randomized controlled trials. J Clin Psychiatry. 2007;68(10):1461-71. |
| Miller LK, Chester R, Jerosch-Herold C. Effects of sensory reeducation programs on functional hand sensibility after median and ulnar repair: a systematic review. J Hand Ther. 2012;25(3):297-306 |
| Parr AT, Manchikanti L, Hameed H, Conn A, Manchikanti KN, Benyamin RM, Diwan S, Singh V, Abdi S. Caudal epidural injections in the management of chronic low back pain: a systematic appraisal of the literature. Pain Physician. 2012;15(3):E159-98. |
| Raman J, MacDermid JC, Grewal R. Effectiveness of different methods of resistance exercises in lateral epicondylosis--a systematic review. J Hand Ther. 2012;25(1):5-25. |
| Schroter S, Glasziou P, Heneghan C. Quality of descriptions of treatments: a review of published randomised controlled trials. BMJ Open. 2012;2(6). pii: e001978. |
| Singh BB, Khorsan R, Vinjamury SP, Der-Martirosian C, Kizhakkeveettil A, Anderson TM. Herbal treatments of asthma: a systematic review. J Asthma. 2007;44(9):685-98. |
| Singh BB, Vinjamury SP, Der-Martirosian C, Kubik E, Mishra LC, Shepard NP, Singh VJ, Meier M, Madhu SG. Ayurvedic and collateral herbal treatments for hyperlipidemia: a systematic review of randomized controlled trials and quasi-experimental designs. Altern Ther Health Med. 2007;13(4):22-8. |
| Trabulsi NH, Patakfalvi L, Nassif MO, Turcotte RE, Nichols A, Meguerditchian AN. Hyperthermic isolated limb perfusion for extremity soft tissue sarcomas: systematic review of clinical efficacy and quality assessment of reported trials. J Surg Oncol. 2012;106(8):921-8. |
| Van Vijven JP, Luijsterburg PA, Verhagen AP, van Osch GJ, Kloppenburg M, Bierma-Zeinstra SM. Symptomatic and chondroprotective treatment with collagen derivatives in osteoarthritis: a systematic review. Osteoarthritis Cartilage. 2012;20(8):809-21. |
| Verbeek J, Martimo KP, Karppinen J, Kuijer PP, Takala EP, Viikari-Juntura E. Manual material handling advice and assistive devices for preventing and treating back pain in workers. Cochrane Database Syst Rev. 2011 Jun 15;(6):CD005958. |
| Wedlake LJ, Shaw C, Whelan K, Andreyev HJ. Systematic review: the efficacy of nutritional interventions to counteract acute gastrointestinal toxicity during therapeutic pelvic radiotherapy. Aliment Pharmacol Ther. 2013;37(11):1046-56. |

| **Main reason for exclusion:**  **The study used an instrument that was not a quality tool (n = 11)** |
| --- |
| Gibson G, Jurasic MM, Wehler CJ, Jones JA. Supplemental fluoride use for moderate and high caries risk adults: a systematic review. J Public Health Dent. 2011;71(3):171-84. |
| Glasgow RE, Gaglio B, Bennett G, Jerome GJ, Yeh HC, Sarwer DB, Appel L, Colditz G, Wadden TA, Wells B. Applying the PRECIS criteria to describe three effectiveness trials of weight loss in obese patients with comorbid conditions. Health Serv Res. 2012;47(3 Pt 1):1051-67 |
| Imdad A, Yakoob MY, Sudfeld C, Haider BA, Black RE, Bhutta ZA. Impact of vitamin A supplementation on infant and childhood mortality. BMC Public Health. 2011;11 Suppl 3:S20 |
| Ishaque S, Yakoob MY, Imdad A, Goldenberg RL, Eisele TP, Bhutta ZA. Effectiveness of interventions to screen and manage infections during pregnancy on reducing stillbirths: a review. BMC Public Health. 2011;11 Suppl 3:S3. |
| Koppenaal T, Linmans J, Knottnerus JA, Spigt M. Pragmatic vs. explanatory: an adaptation of the PRECIS tool helps to judge the applicability of systematic reviews for daily practice. J Clin Epidemiol. 2011;64(10):1095-101 |
| Laws RA, St George AB, Rychetnik L, Bauman AE. Diabetes prevention research: a systematic review of external validity in lifestyle interventions. Am J Prev Med. 2012;43(2):205-14. |
| Matteson KA, Abed H, Wheeler TL 2nd, Sung VW, Rahn DD, Schaffer JI, Balk EM; Society of Gynecologic Surgeons Systematic Review Group. A systematic review comparing hysterectomy with less-invasive treatments for abnormal uterine bleeding. J Minim Invasive Gynecol. 2012;19(1):13-28 |
| Pearson M, Peters J. Outcome reporting bias in evaluations of public health interventions: evidence of impact and the potential role of a study register. J Epidemiol Community Health. 2012;66(4):286-9. |
| Selby P, Brosky G, Oh PI, Raymond V, Ranger S. How pragmatic or explanatory is the randomized, controlled trial? The application and enhancement of the PRECIS tool to the evaluation of a smoking cessation trial. BMC Med Res Methodol. 2012;12:101. |
| Sengupta S, Banks B, Jonas D, Miles MS, Smith GC. HIV interventions to reduce HIV/AIDS stigma: a systematic review. AIDS Behav. 2011;15(6):1075-87. |
| Yuan LP, Zhou X, Li HJ, Wei JY, Ma LM. Aerosol inhalation of sodium houttuyfonate injection: a systematic review. Chinese Journal of New Drugs 2011;20(3):284-8 |

| **Main reason for exclusion:**  **The study used a tool developed for single review purposes (n = 8)** |
| --- |
| Boschen K, Gargaro J, Gan C, Gerber G, Brandys C. Family interventions after acquired brain injury and other chronic conditions: a critical appraisal of the quality of the evidence. NeuroRehabilitation. 2007;22(1):19-41 |
| Camacho-Miñano MJ, LaVoi NM, Barr-Anderson DJ. Interventions to promote physical activity among young and adolescent girls: a systematic review. Health Educ Res. 2011;26(6):1025-49 |
| Daley DJ, Myint PK, Gray RJ, Deane KH. Systematic review on factors associated with medication non-adherence in Parkinson's disease. Parkinsonism Relat Disord. 2012;18(10):1053-61 |
| de Vries AC, Besselink MG, Buskens E, Ridwan BU, Schipper M, van Erpecum KJ, Gooszen HG. Randomized controlled trials of antibiotic prophylaxis in severe acute pancreatitis: relationship between methodological quality and outcome. Pancreatology. 2007;7(5-6):531-8 |
| El Baz N, Middel B, van Dijk JP, Oosterhof A, Boonstra PW, Reijneveld SA. Are the outcomes of clinical pathways evidence-based? A critical appraisal of clinical pathway evaluation research. J Eval Clin Pract. 2007;13(6):920-9 |
| Guo X, Zhou B, Nishimura T, Teramukai S, Fukushima M. Clinical effect of qigong practice on essential hypertension: a meta-analysis of randomized controlled trials. J Altern Complement Med. 2008;14(1):27-37 |
| Khan RJ, Carey Smith RL. Surgical interventions for treating acute Achilles tendon ruptures. Cochrane Database Syst Rev. 2010 Sep 8;(9):CD003674. |
| Liu SS, Togioka BM, Hurley RW, Vu CM, Hanna MN, Murphy JD, Wu CL. Methodological quality of randomized controlled trials of postoperative epidural analgesia: validation of the Epidural Analgesia Trial Checklist as a specific instrument to evaluate methodology. Reg Anesth Pain Med. 2010;35(6):549-55. |

| **Main reason for exclusion:**  **The study was animal research (n = 4)** |
| --- |
| Bucher O, Farrar AM, Totton SC, Wilkins W, Waddell LA, Wilhelm BJ, McEwen SA, Fazil A, Rajić A. A systematic review-meta-analysis of chilling interventions and a meta-regression of various processing interventions for Salmonella contamination of chicken. Prev Vet Med. 2012;103(1):1-15 |
| Simoneit C, Heuwieser W, Arlt S. Evidence-based medicine in bovine, equine and canine reproduction: quality of current literature. Theriogenology. 2011;76(6):1042-50 |
| Simoneit C, Heuwieser W, Arlt SP. Inter-observer agreement on a checklist to evaluate scientific publications in the field of animal reproduction. J Vet Med Educ. 2012;39(2):119-27 |
| Snedeker KG, Canning P, Totton SC, Sargeant JM. Completeness of reporting in abstracts from clinical trials of pre-harvest interventions against foodborne pathogens. Prev Vet Med. 2012;104(1-2):15-22 |

| **Main reason for exclusion:**  **The study did not focus on a particular tool (n = 2)** |
| --- |
| Dechartres A, Charles P, Hopewell S, Ravaud P, Altman DG. Reviews assessing the quality or the reporting of randomized controlled trials are increasing over time but raised questions about how quality is assessed. J Clin Epidemiol. 2011;64(2):136-44 |
| Whelan AM, Jurgens TM, Lord L. Evaluating the quality of randomized controlled trials that examine the efficacy of natural health products: a systematic review of critical appraisal instruments. Evid Based Complement Alternat Med. 2009;6(4):441-8 |

| **Main reason for exclusion:**  **Information on the name of the scale was not provided (n = 1)** |
| --- |
| Parrish AM, Okely AD, Stanley RM, Ridgers ND. The effect of school recess interventions on physical activity: a systematic review. Sports Med. 2013;43(4):287-99 |
