# Supplementary material for: Inconsistency in the items included in tools used in general health research and physical therapy to evaluate the methodological quality of randomized controlled trials: a descriptive analysis
Source: BMC Med Res Methodol. 2013 Sep 17;13:116. doi: 10.1186/1471-2288-13-116 (PMC3848693; doi:10.1186/1471-2288-13-116)
Supplement: Additional file 5 — Heath Sciences Tools and items to Measure Methodological Quality of RCTs. [file 1471-2288-13-116-S5.doc]

**(Additional file 5). Heath Sciences Tools and items to Measure Methodological Quality of RCTs**

|  |  |  |  |  |  |  |  |  |  |  |  |  |  |  |  |  |  |  |  |  |  |  |  |  |
| --- | --- | --- | --- | --- | --- | --- | --- | --- | --- | --- | --- | --- | --- | --- | --- | --- | --- | --- | --- | --- | --- | --- | --- | --- |
| **Items included in the tools** | **Chalmers** | **Consort** | **Cho** | **Detsky** | **Jadad** | **Reisch** | **Sindhu** | **Andrew** | **OPVS** | **Arrive** | **Yates** | **Imperiale** | **Balas** | **Downs** | **Nguyen** | **RCT-PQRS** | **RCT_NPS** | **CCDAN** | **CLEAR NPT** | **Total items**  **(n)** | **FREQ %** | **R*** | **C*** | **Threats to**  **Validity**  **and precision** |
| **INTRODUCCION, OBJECTIVES, AND DESIGN** |  |  |  |  |  |  |  |  |  |  |  |  |  |  |  |  |  |  |  |  |  |  |  |  |
| 1.Title consistent with study purpose |  |  |  |  |  | **X** |  |  |  |  |  |  |  |  |  |  |  |  |  | 1 | 5.3 | X |  | None |
| 2. Title identify the study as randomized |  | X |  |  |  |  |  |  |  |  |  |  |  |  |  |  |  |  |  | 1 | 5.3 | X |  | None |
| 3. Abstract presented in a structured format |  | X |  |  |  |  |  |  |  |  |  |  |  |  |  |  |  |  |  | 1 | 5.3 | X |  | None |
| 4.Study question/hypothesis/purpose described and well defined |  | X | X |  |  | X | X | X |  | X |  |  |  | X | X |  | X | X |  | 10 | 52.3 | X |  | None |
| 5. Study design appropriate to answer study question |  |  | X |  |  |  | X | X |  | X |  |  |  |  | X |  |  |  |  | 5 | 26.3 |  | X | Multiple Biases |
| 6.Use of a well-matched Control Group |  |  |  |  |  |  |  |  |  |  | X |  |  |  |  |  |  |  |  | 1 | 5.3 |  | X | Selection Bias |
| 7. A priori relevant hypotheses that justify comparison group (s**)** |  |  |  |  |  |  |  |  |  |  |  |  |  |  |  | X |  |  |  | 1 | 5.3 | X |  | None |
| 8.Known confounders accounted for by study design |  |  | X |  |  |  |  |  |  |  |  |  |  |  | X |  |  |  |  | 2 | 10.5 |  | X | Multiple Biases |
| **PATIENT SELECTION (INCLUSION AND EXCLUSION AND DESCRIPTION OF SUBJECTS)** |  |  |  |  |  |  |  |  |  |  |  |  |  |  |  |  |  |  |  |  |  |  |  |  |
| 9.Inclusion and exclusion criteria clearly defined | X | X | X | X |  | X |  | X |  | X | X | X | X |  | X | X | X | X |  | 14 | 73.7 | X |  | Selection Bias (Ascertainment bias) |
| 10.Baseline comparability (group equivalence, homogeneity) / Control and experimental groups matched at baseline (ex. Age, gender, weight, etc.). Baseline Comparability | X | X |  |  |  | X | X |  |  |  | X | X |  |  |  |  |  | X |  | 7 | 36.8 |  | X | Selection Bias  (Ascertainment bias) |
| 11.Trial site/cases and control recruited from the same population |  |  |  |  |  |  |  |  |  |  |  |  |  | X |  | X |  |  |  | 2 | 10.5 |  | X | Selection Bias  (Ascertainment bias) |
| 12.Cases and control recruited over the same period of time |  |  |  |  |  |  |  |  |  |  |  |  |  | X |  | X |  |  |  | 2 | 10.5 |  | X | Selection Bias  (Ascertainment bias) |
| 13.Subjects represent the entire population |  |  |  |  |  |  |  |  |  |  |  |  | X | X |  |  |  | X |  | 3 | 15.8 |  | X | Selection Bias  (Ascertainment bias) |
| 14.Sample characteristics/ Sample demographics |  | X | X |  |  | X | X |  |  | X | X |  | X | X | X |  |  | X |  | 10 | 52.3 | X |  | Selection Bias  (Ascertainment bias) |
| 15.Clear reporting of the number of subjects excluded from the trial | X |  |  | X |  |  |  | X |  |  |  |  | X |  |  | X |  | X |  | 6 | 31.6 | X |  | Selection Bias  (Ascertainment bias) |
| 16.Number of people approached to participate in the study was reported (subjects screened) | X | X |  |  |  |  |  |  |  |  |  |  |  |  |  | X |  |  |  | 3 | 15.8 | X |  | Selection Bias  (Ascertainment bias) |
| 17.Number of patients that refused to participate was reported | X |  |  | X |  | X |  |  |  |  |  |  |  |  |  |  |  | X |  | 4 | 21.1 | X |  | Selection Bias  (Ascertainment bias) |
| 18.Documentation or demonstration of reliability of diagnostic methodology |  |  |  |  |  |  |  |  |  |  |  |  |  |  |  | X |  |  |  | 1 | 5.3 |  | X | None |
| 19.Description of relevant co morbidities |  |  |  |  |  |  |  |  |  |  |  |  |  |  |  | X |  |  |  | 1 | 5.3 | X |  | Selection Bias |
| 20.Subjects appropriate to study questions |  |  | X |  |  | X |  |  |  |  |  |  |  |  |  |  |  |  |  | 2 | 10.5 |  | X | Selection Bias  (Ascertainment bias) |
| 21.Subjects randomly selected from the target population |  |  | X |  |  |  |  |  |  |  |  |  |  |  |  |  |  |  |  | 1 | 5.3 |  | X | Selection Bias |
| 22. Equality of study groups based on appropriate prognostic factors (ex. Prognostic factors specific to a certain condition, therapy, etc.)/or confounders | X |  |  |  |  |  |  |  |  |  |  |  |  | X |  |  |  |  |  | 2 | 10.5 |  | X | Selection bias  (Ascertainment bias) |
| 23.Time period of subject selection (example: subjects evaluated prospectively and/or selected prior to treatment and evaluation versus subjects evaluated retrospectively and/or selected once study has already been planned or after treatment is completed) |  |  |  |  |  | X |  |  |  |  |  |  |  |  |  |  |  |  |  | 1 | 5.3 |  | X | Selection bias  (different between groups) |
| 24.Sample Representative and source of subjects described | X |  |  |  |  |  |  |  |  |  |  |  |  |  |  |  |  | X |  | 2 | 10.5 | X | X | Selection Bias  (Ascertainment bias) |
| 25. Are prognostic variables by treatment and control group described? |  | X |  |  |  |  |  |  |  |  |  |  |  |  |  |  |  |  |  | 1 | 5.3 | X |  | Selection bias |
| 26. Description of confounders |  |  |  |  |  |  |  |  |  |  |  |  |  |  | X |  |  |  |  | 1 | 5.3 | X |  | Selection bias |
| **ASSIGNMENT, RANDOMIZATION, AND ALLOCATION CONCEALMENT** |  |  |  |  |  |  |  |  |  |  |  |  |  |  |  |  |  |  |  |  |  |  |  |  |
| 27.Study described as randomized (randomization assignment to treatment groups) | X | X | X | X | X | X | X |  |  |  | X |  |  |  |  | X | X | X |  | 11 | 57.9 | X |  | Selection Bias |
| 28.Description of randomization process reported and appropriate | X | X | X | X | X | X | X | X |  |  |  |  | X | X |  |  | X |  | X | 12 | 63.2 | X | X | Selection Bias |
| 29.Randomized intervention assignment concealed from patients and care staff |  |  |  |  |  |  |  |  |  |  |  |  |  | X |  |  | X | X | X | 4 | 21.1 |  | X | Selection Bias |
| 30. Method of randomization blinded (allocation concealment) reported | **X** | X |  | X |  |  | X |  |  |  | X |  |  |  |  |  | X |  | X | 7 | 36.8 | X |  | Selection Bias |
| 31.Control of bias in regards to treatment assignment |  |  |  | X |  |  | X |  |  |  | X |  |  |  |  |  |  |  |  | 2 | 15.8 |  | X | Selection Bias |
| 32.Testing of randomization performed | X |  |  |  |  |  |  |  |  |  |  |  | X |  |  |  |  |  |  | 2 | 10.5 |  | X | Selection Bias |
| 33. Time of assignment described |  | X |  |  |  |  |  |  |  |  |  |  |  |  |  |  |  |  |  | 1 | 5.3 | X |  | None |
| 34. Is the number of randomized patients reported for each comparison group |  | X |  |  |  |  |  |  |  |  |  |  |  |  |  |  |  |  |  | 1 | 5.3 | X |  | Selection Bias/Attrition Bias |
| **BLINDING** |  |  |  |  |  |  |  |  |  |  |  |  |  |  |  |  |  |  |  |  |  |  |  |  |
| 35.Study described as double blind |  |  |  |  | X |  |  | X | X |  |  |  |  |  |  |  |  |  |  | 3 | 15.8 | X |  | Performance Bias/Detection Bias |
| 36.Blinding of Investigator/Assessor | X |  | X | X |  | X | X |  |  | X |  |  | X | X | X | X |  | X | X | 12 | 63.2 | X | X | Detection Bias |
| 37.Blinding of Subjects | X |  | X |  |  | X | X | X |  |  |  |  | X | X |  |  |  | X | X | 9 | 47.4 | X | X | Performance Bias/Detection Bias  (self-reported outcome assessment) |
| 38.Blinding of Therapists | X |  |  | X |  | X | X | X |  |  |  |  | X |  |  |  |  |  | X | 7 | 36.8 | X | X | Performance Bias |
| 39.Blinding of the outcome (results) | X |  |  |  | X |  |  |  |  |  |  |  |  |  |  |  |  |  |  | 2 | 10.5 | X | X | Detection Bias |
| 40.Testing of blinding | X |  |  |  |  |  |  |  |  |  |  |  |  |  |  |  |  |  |  | 1 | 5.3 |  | X | Performance Bias/Detection Bias |
| 41.Blinding of statistician, if applicable | X |  |  |  |  |  |  |  |  |  |  |  |  |  |  |  |  |  |  | 1 | 5.3 | X | X | Detection Bias |
| 42. Study was blinded**]** |  |  |  |  |  |  |  |  | X |  |  |  |  |  |  |  | X |  |  | 2 | 10.5 |  | X | Performance Bias/Detection Bias |
| 43. Was method of double blinding appropriate |  |  |  |  | X |  |  |  |  |  |  |  |  |  |  |  |  |  |  | 1 | 5.3 |  | X | Performance Bias/Detection Bias |
| 44. Mechanism of blinding described |  | X |  |  |  |  |  |  |  |  |  |  |  |  |  |  |  |  |  | 1 | 5.3 | X |  | Performance Bias/Detection Bias |
| 45. Reasons given as to why assignment was not blinded |  |  |  |  |  |  | X |  |  |  |  |  |  |  |  |  |  |  |  | 1 | 5.3 | X |  | None |
| 46. Discussion of bias resulting from non-blinding assessment |  |  |  |  |  |  | X |  |  |  |  |  |  |  |  |  |  |  |  | 1 | 5..3 | X |  | None |
| **INTERVENTIONS** |  |  |  |  |  |  |  |  |  |  |  |  |  |  |  |  |  |  |  |  |  |  |  |  |
| 47.Treatment protocol adequately described for the treatment and control groups (ex. frequency, intensity) | X | X |  | X |  | X |  | X |  |  | X |  | X | X |  | X | X | X | X | 12 | 63.2 | X |  | Performance Bias |
| 48.Cointerventions avoided |  |  |  |  |  |  |  |  |  |  |  | X |  |  |  |  |  |  |  | 1 | 5.3 |  | X | Performance Bias/Contamination Bias  ( if different between groups) |
| 49.Equal use of co-therapies ( co-interventions comparable) |  |  |  |  |  |  |  |  |  |  |  | X |  |  |  |  |  |  | X | 2 | 10.5 |  | X | Performance Bias/Contamination Bias ( if different between groups) |
| 50.Control regiment appearance and taste standardized and not different than that of the actual treatment (if applicable) / treatments Comparable / Control identical of Treatment group except for treatment being administered | X |  |  |  |  | X |  |  |  |  |  | X |  |  |  |  | X |  |  | 4 | 21.1 |  | X | Performance Bias/Contamination Bias  ( if different between groups) |
| 51.Testing of subject compliance to treatment protocol /report of compliance | X |  |  |  |  | X | X |  |  |  | X |  |  | X |  |  | X | X | X | 8 | 42.1 | X | X | Performance Bias/Compliance Bias  ( if different between groups) |
| 52.Biological equivalent performed if applicable (example: if using drug treatment, testing should be done to ensure that the drug is in its active form in the patient’s body) | X |  |  |  |  |  |  |  |  |  |  |  |  |  |  |  |  |  |  | 1 | 5.3 |  | X | Performance Bias/Contamination Bias  ( if different between groups) |
| 53.Treatment duration |  |  |  |  |  |  |  |  |  |  | X |  |  |  |  |  |  |  |  | 1 | 5.3 | X |  | None |
| 54.Therapist training and level of experience in the treatment(s) under investigation |  |  |  |  |  |  |  |  |  |  | X |  |  |  |  | X |  |  | X | 3 | 15.8 | X | X | Performance Bias/Contamination Bias  ( if different between groups) |
| 55.Treatment Manual that describes the components of treatment |  |  |  |  |  |  |  |  |  |  | X |  |  |  |  |  |  |  |  | 1 | 5.3 | X |  | None |
| 56.Intervention representative of that in source population |  |  |  |  |  |  |  |  |  |  |  |  |  | X |  |  |  |  |  | 1 | 5.3 |  | X | None |
| 57.Treatment given to patients was reasonable and appropriate to answer the study question |  |  |  |  |  | X |  |  |  |  |  | X |  |  |  |  |  |  |  | 2 | 10.5 |  | X | None |
| 58.Description of concurrent treatments (e.g. medication) allowed and administered during course of the study |  |  |  |  |  |  |  |  |  |  |  |  |  |  |  | X |  |  |  | 1 | 5.3 | X |  | Performance Bias/Contamination Bias |
| 59.Method to demonstrate that treatment being studied is treatment being delivered (only satisfied by supervision if transcripts or tapes are explicitly reviewed) |  |  |  |  |  |  |  |  |  |  |  |  |  |  |  | X |  |  |  | 1 | 5.3 |  | X | Performance Bias |
| 60.Therapist supervision while treatment is being provided |  |  |  |  |  |  |  |  |  |  |  |  |  |  |  | X |  |  |  | 1 | 5.3 |  | X | Performance Bias |
| 61.Appropriate consideration of therapist and site effects |  |  |  |  |  |  |  |  |  |  |  |  |  |  |  | X |  |  |  | 1 | 5.3 |  | X | Performance Bias/Contamination Bias /Differential Maturing Bias |
| 62.Balance of allegiance to types of treatment by practitioners |  |  |  |  |  |  |  |  |  |  |  |  |  |  |  | X |  |  |  | 1 | 5.3 |  | X | Performance Bias |
| 63. Adherence to study protocol |  |  |  |  |  |  | X |  |  |  |  |  |  |  |  |  |  |  |  | 1 | 5.3 |  | X | Performance Bias/Compliance bias |
| **ATTRITION, FOLLOW UP AND PROTOCOL DEVIATION** |  |  |  |  |  |  |  |  |  |  |  |  |  |  |  |  |  |  |  |  |  |  |  |  |
| 64.Description of withdraws and dropouts | X | X | X |  | X | X | X |  | X |  | X |  | X |  | X | X |  | X |  | 12 | 63.2 | X |  | Attrition Bias |
| 65.Reasons for dropouts |  |  | X |  | X |  |  |  |  |  |  |  |  |  |  |  |  | X |  | 3 | 15.8 | X |  | Attrition Bias |
| 66.Procedures established to minimize loss of subjects |  |  |  |  |  | X |  |  |  |  |  |  |  |  | X |  |  |  |  | 2 | 10.5 |  | X | Attrition Bias |
| 67.Protocol deviations described for each comparison group |  | X |  |  |  |  |  |  |  |  |  |  |  |  |  |  |  |  |  | 1 | 5.3 | X |  | Attrition Bias |
| 68.Evidence that reporting of attrition followed CONSORT guidelines |  |  |  |  |  |  |  |  |  |  | X |  |  |  |  |  |  |  |  | 1 | 5.3 | X |  | None |
| 69. Characteristics of patients lost to follow up described |  |  |  |  |  |  |  |  |  |  |  |  |  | X |  |  |  |  |  | 1 | 5..3 | X |  | Attrition Bias  (If different between groups) |
| 70.Patient follow-up details reported |  |  |  |  |  |  | X |  |  |  | X |  |  |  |  |  |  |  |  | 2 | 10.5 | X |  | Attrition Bias |
| 71. Assessment of long-term post termination outcome |  |  |  |  |  |  |  |  |  |  |  |  |  |  | X | X |  |  |  | 2 | 10.5 |  | X | Attrition Bias |
| 72. Were withdrawals and lost to follow-up the same in each randomized group |  | X |  |  |  | X |  |  |  |  |  |  |  |  |  |  |  |  | X | 1 | 5.3 |  | X | Attrition Bias |
| 73.Was follow-up schedule the same in each group |  |  |  |  |  |  |  |  |  |  |  |  |  |  |  |  |  |  | X | 1 | 5.3 |  | X | Attrition Bias |
| **OUTCOMES** |  |  |  |  |  |  |  |  |  |  |  |  |  |  |  |  |  |  |  |  |  |  |  |  |
| 74.Description of outcome measures /criteria for measuring outcomes |  | X |  | X |  | X | X |  | X |  | X |  | X | X | X |  |  | X |  | 10 | 52.3 | X |  | Reporting Bias |
| 75.Numeric table of effect variables |  |  |  |  |  |  |  |  |  |  |  |  | X |  |  |  |  |  |  | 1 | 5.3 | X |  | Reporting Bias |
| 76.Validity reported for main outcome measures |  |  |  |  |  |  | X |  |  |  | X |  |  | X |  | X |  | X |  | 5 | 26.3 | X | X | Information Bias |
| 77.Responsiveness/ Sensitivity to change reported |  |  |  |  |  |  |  |  |  |  | X |  |  |  |  |  |  |  |  | 1 | 5.3 | X | X | Information Bias |
| 78.Number of examiners reported |  |  |  |  |  |  |  |  |  |  |  |  |  |  | X |  |  |  |  | 1 | 5.3 | X |  | None |
| 79.Reliability reported for main outcome measures |  |  |  |  |  |  | X |  |  | X | X |  |  | X | X | X |  |  |  | 6 | 31.6 | X | X | Information Bias |
| 80.Use of objective outcome measures |  |  |  | X |  |  | X |  |  |  |  |  |  |  |  |  |  |  |  | 2 | 10.5 |  | X | Information Bias |
| 81.Standardized and consistent laboratory procedures and other measurement procedures |  |  |  |  |  | X |  |  |  |  |  |  |  |  |  |  |  |  |  | 1 | 5.3 |  | X | Information Bias |
| 82. Primary Outcome measure(s) specified in advance | X | X |  |  |  |  |  |  |  |  |  |  |  |  |  | X | X | X |  | 5 | 26.3 | X |  | Reporting Bias |
| 83.Minimum important difference for primary outcome reported | X | X |  |  |  | X |  |  |  |  |  |  |  |  |  |  |  |  |  | 3 | 15.8 | X |  | Reporting Bias |
| 84.Relevant outcomes to the research question |  |  |  |  |  |  |  |  | X |  |  |  |  |  |  |  |  |  |  | 1 | 5.3 |  | X | Information Bias |
| 85.Description of secondary outcomes |  |  |  |  |  |  |  |  |  |  |  |  | X |  |  |  |  |  |  | 1 | 5.3 | X |  | Reporting Bias |
| 86.Tabulation of study events that are employed as endpoints | X |  |  |  |  |  |  |  |  |  |  |  |  |  |  |  |  |  |  | 1 | 5.3 | X |  | Reporting Bias |
| 87.Adjustment for different lengths of follow-up, or time period between intervention and outcome the same for cases and control |  |  |  |  |  |  |  |  |  |  |  |  |  | X |  |  |  |  |  | 1 | 5.3 |  | X | Detection Bias |
| 88.Were specific methods used to avoid systematic differences in outcome assessment ( ascertainment bias) |  |  |  |  |  |  |  |  |  |  | X |  |  |  |  |  |  | X |  | 2 | 10.5 |  | X | Detection Bias |
| **STATISTICAL ANALYSIS** |  |  |  |  |  |  |  |  |  |  |  |  |  |  |  |  |  |  |  |  |  |  |  |  |
| 89.Intention to treat analysis used |  |  |  |  |  |  | X |  | X |  | X |  |  |  |  | X |  | X | X | 6 | 31.6 |  | X | Selection Bias/Attrition Bias/lack of Intention to treat bias |
| 90.Analysis of main effect variables/on major endpoints | X |  |  |  |  |  |  |  |  |  |  |  | X |  |  |  |  |  |  | 2 | 10.5 |  | X | Reporting Bias |
| 91.Analysis of secondary variables |  |  |  |  |  |  |  |  |  |  |  |  | X |  |  |  |  |  |  | 1 | 5.3 |  | X | Reporting Bias |
| 92.Type of statistical test used clearly stated | X | X | X | X |  | X |  |  | X | X | X |  |  |  |  |  |  |  |  | 8 | 42.1 | X |  | None |
| 93.P value and/or confidence intervals reported | X | X | X | X |  |  |  |  |  |  |  |  |  | X |  |  |  | X |  | 6 | 31.6 | X |  | None |
| 94.Actual probability values reported for the main outcome (e.g.: 0.035) | X |  |  |  |  |  |  |  |  |  |  |  |  | X |  |  |  |  |  | 2 | 10.5 | X |  | None |
| 95.Appropriate statistical analysis used (e.g. use of Bonferroni correction, longitudinal data analysis, adjustment only for a priori identified confounders ) | X | X | X | X |  | X | X | X | X | X | X |  |  | X | X | X |  | X |  | 14 | 73.7 |  | X | Threats to precision |
| 96.Presentation of data |  |  |  |  |  |  |  |  |  |  |  |  |  |  | X |  |  | X |  | 2 | 10.5 | X |  | Reporting Bias |
| 97.Appropriate interpretation of statistical results |  |  |  |  |  | X |  |  |  |  |  |  |  |  |  |  |  |  |  | 1 | 5.3 |  | X | Statistical Bias |
| 98.Post hoc power calculation performed and confidence intervals reported (if trial is negative)/ Power calculation | X | X | X | X |  |  |  |  |  |  | X |  | X | X |  |  |  | X |  | 8 | 42.1 | X | X | Threats to precision |
| 99.Life table/life series analysis or regression analysis correlation performed if appropriate | X |  |  |  |  |  |  |  |  |  |  |  |  |  |  |  |  |  |  | 1 | 5.3 |  | X | Statistical Bias |
| 100.Statistical handling of withdrawals | X |  |  |  |  |  |  |  |  |  |  |  | X | X | X |  |  |  |  | 4 | 21.1 |  | X | Attrition Bias |
| 101.Side effects reported / examined statistically | X |  |  |  |  |  |  | X |  |  |  |  |  |  |  | X |  |  |  | 3 | 15.8 | X |  | Reporting Bias |
| 102.Retrospective analysis performed | X |  |  |  |  |  |  |  |  |  |  |  | X |  |  |  |  |  |  | 2 | 10.5 |  | X | None |
| 103.Multiple looks considered | X |  |  |  |  |  |  |  |  |  |  |  | X |  |  |  |  |  |  | 2 | 10.5 |  | X | Statistical Bias/  Threats to precision |
| 104.Sample size calculation performed prior to initiation of the study | X |  | X | X |  | X | X |  |  |  | X |  | X |  | X |  | X |  |  | 9 | 47.4 |  | X | None |
| 105.Sample size number reported (as allocated, for each comparison group) |  | X |  |  |  | X | X |  | X |  |  |  |  |  |  |  |  |  |  | 4 | 21.1 | X |  | None |
| 106.Adequate sample size |  |  |  |  |  | X |  |  |  |  | X |  |  |  | X | X |  | X |  | 5 | 26.3 |  | X | Threats to precision |
| 107.Sufficient power to detect a clinically important effect |  |  |  |  |  |  | X |  |  |  |  |  |  | X |  |  |  |  |  | 2 | 10.5 |  | X | Threats to precision |
| 108.Descriptive measures identified and reported |  |  |  |  |  | X | X |  | X |  |  |  | X | X |  |  |  |  |  | 5 | 26.3 | X |  | None |
| 109.Errors in results/statistics identified |  |  |  |  |  | X |  |  |  |  |  |  |  |  |  |  |  |  |  | 1 | 5.3 | X |  | Statistical Bias |
| 110.Statistical analysis completed for all the main/primary outcomes | X |  |  |  |  |  | X |  |  |  |  |  |  |  |  |  |  |  |  | 2 | 10.5 |  | X | Reporting Bias |
| 111.Results reported for all subjects that completed the study |  |  | X |  |  |  |  |  |  |  |  |  |  |  |  |  |  |  |  | 1 | 5.3 | X |  | Selection Bias/Attrition/Reporting |
| 112.Known confounders accounted for by statistical analysis /baseline differences accounted in analysis |  |  | X |  |  |  | X |  |  |  |  |  |  | X | X |  |  | X |  | 5 | 26.3 |  | X | Selection bias/ Threats to precision |
| 113.Data on possible adverse effects | X |  |  |  |  | X |  |  |  |  |  |  |  | X |  | X |  | X |  | 5 | 26.3 | X |  | Reporting Bias |
| 114.Indication of analysis that had not been planned at the onset of the study |  |  |  |  |  |  |  |  |  |  |  |  |  | X |  |  |  |  |  | 1 | 5.3 | X |  | Reporting Bias |
| 115. Are stopping rules described |  | X |  |  |  |  |  |  |  |  |  |  |  |  |  |  |  |  |  | 1 | 5.3 | X |  | Threats to precision /Statistical Bias |
| 116.Results stated in absolute numbers |  | X |  |  |  |  |  |  |  |  |  |  |  |  |  |  |  |  |  | 1 | 5.3 | X |  | None |
| 117.Number of patients analyzed reported for each group |  | X |  |  |  |  |  |  |  |  |  |  |  |  |  |  |  |  |  | 1 | 5.3 | X |  | Reporting Bias |
| 118.Clinical and statistical significance reported |  |  |  |  |  |  | X |  |  |  |  |  |  |  |  |  |  |  |  | 1 | 5.3 | X |  | Reporting Bias |
| 119. Is the Unit of analysis described |  | X |  |  |  |  |  |  |  |  |  |  |  |  |  |  |  |  |  | 1 | 5.3 | X |  | None |
| **MISCELANEOUS** |  |  |  |  |  |  |  |  |  |  |  |  |  |  |  |  |  |  |  |  |  |  |  |  |
| 120. Start and stop dates of study reported. The duration of the study was stated | X |  |  |  |  |  |  |  |  |  |  |  |  |  |  |  | X |  |  | 2 | 10.5 | X |  | None |
| 121.Timing of study events reported | X | X |  |  |  |  |  |  |  |  |  |  | X |  |  |  |  |  |  | 3 | 15.8 | X |  | None |
| 122.Study conclusion supported by the findings of the study (statistical results) |  |  | X |  |  |  | X | X |  |  |  |  |  |  | X | X |  | X |  | 6 | 31.6 | X |  | None |
| 123.Informed consent obtained /prior to randomization [Sindhu] |  |  |  |  |  | X | X | X |  |  |  |  |  |  |  |  |  |  |  | 3 | 15.8 | X |  | None |
| 124.If applicable, cost effectiveness discussed |  |  |  |  |  | X |  |  |  |  |  |  |  |  |  |  |  |  |  | 1 | 5.3 | X |  | None |
| 125.Appropriate duration of trial including follow-up |  |  |  |  |  |  |  |  |  |  |  |  |  |  |  |  |  | X |  | 1 | 5.3 |  | X | None |
| 126.Declaration of Interest (source of funding) | X |  |  |  |  | X |  |  |  |  |  |  |  |  |  |  |  | X |  | 3 | 15.8 | X |  | Reporting Bias |
| 127.Study accounted for bias using methods other than blinding |  |  | X |  |  |  |  |  |  |  |  |  |  |  |  |  |  |  |  | 1 | 5.3 |  | X | Performance Bias |
| 128. Description of the site (e.g. medical specialty, inpatient, outpatients) |  |  |  |  |  |  |  |  |  |  |  |  | X |  |  |  |  |  |  | 1 | 5.3 | X |  | None |
| 129. Main findings clearly described |  |  |  |  |  |  |  |  |  |  |  |  |  | X |  |  |  |  |  | 1 | 5.3 | X |  | Reporting Bias |
| 130.data collected prospectively |  |  |  |  |  | X |  |  |  |  |  |  |  |  |  |  |  |  |  | 1 | 5.3 |  | X | Multiple Biases |

**R*= reporting item**

**C*= conduct item**

**RCT-PQRS:** the Randomized Controlled Trial Psychotherapy Quality Rating Scale

**RCT-NPS:** the Randomized Controlled Trial -Natural Products Tool

**CCDAN:** the Cochrane Collaboration Depression, Anxiety, And Neurosis tool **or PQRS:** Psychotherapy Quality Rating Scale

**CLEAR NPT**: A checklist to evaluate a report of a nonpharmacological trial
