# Supplementary material for: Inconsistency in the items included in tools used in general health research and physical therapy to evaluate the methodological quality of randomized controlled trials: a descriptive analysis
Source: BMC Med Res Methodol. 2013 Sep 17;13:116. doi: 10.1186/1471-2288-13-116 (PMC3848693; doi:10.1186/1471-2288-13-116)
Supplement: Additional file 6 — Tools and Items to Assess Quality of RCTs in Physical Therapy. [file 1471-2288-13-116-S6.doc]

**(**Additional file **6). Tools and Items to Assess Quality of RCTs in Physical Therapy**

| **Items included in the scales** | Item Number | Jadad | Maastricht | Delphi | Van Tulder  (2003) | Maastricht-  Amsterdam | PeDro | Bizzini | **Total items (n)** | **FREQ %** | **Reporting** | **Conduction** | **Threats to validity and precision** |
| --- | --- | --- | --- | --- | --- | --- | --- | --- | --- | --- | --- | --- | --- |
| **PATIENT SELECTION (INCLUSION AND EXCLUSION AND DESCRIPTION OF SUBJECTS)** |  |  |  |  |  |  |  |  |  |  |  |  |  |
| Inclusion criteria clearly defined/eligibility criteria specified | 1 |  | X | X |  | X | X | X | 5 | 71.4 | X |  | Selection Bias. (Ascertainment Bias) |
| Exclusion criteria defined | 2 |  | X |  |  |  |  | X | 2 | 28.6 | X |  | Selection Bias. (Ascertainment Bias) |
| Restriction to a homogeneous study population | 3 |  | X |  |  |  |  |  | 1 | 14.3 |  | X | Selection Bias. (Ascertainment Bias) |
| Baseline comparability (group equivalence, homogeneity) regarding the most important prognostic indicators | 4 |  | X | X | X | X | X | X | 6 | 85.7 |  | X | Selection Bias. |
| **ASSIGNMENT, RANDOMIZATION, AND ALLOCATION CONCEALMENT** |  |  |  |  |  |  |  |  |  |  |  |  |  |
| Study described as randomized | 5 | X |  |  |  |  | X | X | 3 | 42.9 | X |  | Selection Bias. |
| Randomization method performed | 6 |  | X | X |  | X |  | X | 4 | 57.1 |  | X | Selection Bias. |
| Method of randomization described | 7 | X |  |  |  |  | X | X | 3 | 42.9 | X |  | Selection Bias. |
| Method of randomization appropriate | 8 | X | X |  | X | X | X |  | 5 | 71.4 |  | X | Selection Bias. |
| Method of randomization concealed | 9 |  | X | X | X | X | X |  | 5 | 71.4 |  | X | Selection Bias. |
| **BLINDING** |  |  |  |  |  |  |  |  |  |  |  |  |  |
| Study described as double blind | 10 | X |  |  |  |  |  |  | 1 | 14.3 | X |  | Performance Bias/Detection Bias (outcome assessment) |
| Method of blinding described | 11 | X |  |  |  |  |  |  | 1 | 14.3 | X |  | Performance Bias/Detection Bias (outcome assessment) |
| Method of blinding appropriate | 12 | X |  |  |  |  |  |  | 1 | 14.3 |  | X | Performance Bias/Detection Bias (outcome assessment) |
| Blinding of Investigator/Assessor | 13 |  | X | X | X | X | X | X | 6 | 85.7 |  | X | Detection Bias (outcome assessment) |
| Observer blinding evaluated and successful | 14 |  | X |  |  |  |  |  | 1 | 14.3 |  | X | Detection Bias  (outcome assessment) |
| Blinding of Subjects/patients | 15 |  | X | X | X | X | X |  | 5 | 71.4 |  | X | Performance Bias/Detection Bias  (self-reported outcome assessment) |
| Patient blinding evaluated and successful | 16 |  | X |  |  |  |  |  | 1 | 14.3 |  | X | Performance Bias/Detection Bias  (self-reported outcome assessment) |
| Blinding of Therapists/care provider | 17 |  | X | X | X | X | X |  | 5 | 71.4 |  | X | Performance Bias |
| Therapist blinding evaluated and successful | 18 |  | X |  |  |  |  |  | 1 | 14.3 |  | X | Performance Bias |
| Blinding of the outcome (Data Analyst) | 19 |  | X |  |  |  |  |  | 1 | 14.3 |  | X | Detection Bias |
| **INTERVENTIONS** |  |  |  |  |  |  |  |  |  |  |  |  |  |
| Treatment protocol adequately described for the treatment group regarding Type of intervention, duration of each intervention, frequency, intensity and dosage | 20 |  | X |  |  | X |  | X | 3 | 42.9 | X |  | Performance Bias |
| Treatment protocol adequately described for the Control group regarding Type of intervention, duration of each intervention, frequency, intensity and dosage | 21 |  | X |  |  | X |  |  | 2 | 28.6 | X |  | Performance Bias |
| Control adequate (Presence of a control group) | 22 |  |  |  |  |  |  | X | 1 | 14.3 |  | X | Performance Bias |
| Placebo adequate (Presence of a placebo group) | 23 |  |  |  |  |  |  | X | 1 | 14.3 |  | X | Performance Bias |
| Co-interventions avoided/or comparable | 24 |  | X |  | X | X |  | X | 4 | 57.1 |  | X | Performance Bias/Contamination Bias |
| Co-interventions reported for each group separately | 25 |  |  |  |  | X |  |  | 1 | 14.3 | X |  | Performance Bias/Contamination Bias |
| Testing of subject compliance to treatment protocol (report of compliance) | 26 |  | X |  |  |  |  |  | 1 | 14.3 | X | X | Performance Bias/Compliance bias |
| Compliance acceptable in all groups (80% of treatment received) | 27 |  |  |  | X | X |  |  | 2 | 28.6 |  | X | Performance Bias/Compliance bias |
| **ATTRITION, FOLLOW UP AND PROTOCOL DEVIATION** |  |  |  |  |  |  |  |  |  |  |  |  |  |
| Report of withdraws and dropouts (rate) | 28 | X | X |  | X | X |  | X | 5 | 71.4 | X |  | Attrition Bias |
| Withdrawal/dropouts rate acceptable (less than 20%) | 29 |  | X (less than 5%) |  | X | X | X (15%) |  | 4 | 57.1 |  | X | Attrition Bias |
| Reasons for withdraws and dropouts reported | 30 | X | X |  |  |  |  | X | 3 | 42.9 | X |  | Attrition Bias |
| Adverse effects described | 31 |  | X |  |  | X |  |  | 2 | 28.6 | X |  | Reporting Bias |
| Patient follow-up details reported | 32 |  | X |  |  |  |  |  | 1 | 14.3 | X |  | Attrition Bias/losses to follow up |
| Follow-up period adequate (Acute: up to 3 months / Chronic: not less than 6 months follow up) | 33 |  | X |  |  |  |  | X | 2 | 28.6 |  | X | Attrition Bias |
| Short follow-up measurement performed | 34 |  |  |  |  | X |  |  | 1 | 14.3 |  | X | Attrition Bias |
| Intermediate-term follow-up performed | 35 |  |  |  |  | X |  |  | 1 | 14.3 |  | X | Attrition Bias |
| Long term follow-up measurement performed | 36 |  |  |  |  | X |  |  | 1 | 14.3 |  | X | Attrition Bias |
| **OUTCOMES** |  |  |  |  |  |  |  |  |  |  |  |  |  |
| Outcome measures described | 37 |  |  |  |  |  |  | X | 1 | 14.3 | X |  | Reporting Bias |
| Relevant outcomes were included (there was a good rationale between intervention and outcome) | 38 |  |  |  |  | X |  | X | 2 | 28.6 |  | X | Information Bias |
| Validity for main outcome measures reported | 39 |  |  |  |  |  |  | X | 1 | 14.3 | X | X | Information Bias |
| Responsiveness for main outcome measures reported | 40 |  |  |  |  |  |  | X | 1 | 14.3 | X | X | Information Bias |
| Reliability for main outcome measures reported | 41 |  |  |  |  |  |  | X | 1 | 14.3 | X | X | Information Bias |
| Use of objective outcome measures (use of measurements that can be scored) | 42 |  | X |  |  |  |  |  | 1 | 14.3 |  | X | Information Bias |
| The timing of the outcome assessment was comparable in all groups (same time) | 43 |  |  |  | X | X |  |  | 2 | 28.6 |  | X | Detection Bias |
| **STATISTICAL ANALYSIS** |  |  |  |  |  |  |  |  |  |  |  |  |  |
| Descriptive measures (point estimates and measures of variability) identified and reported for the primary outcome | 44 |  | X | X |  | X | X | X | 5 | 71.4 | X |  | Reporting Bias |
| Appropriate statistical analysis used | 45 |  | X |  |  |  | X | X | 3 | 42.9 |  | X | Statistical Bias |
| Adequate sample size | 46 |  | X |  |  |  |  | X | 2 | 28.6 |  | X | Threats to precision |
| Sample size described for each group | 47 |  |  |  |  | X |  |  | 1 | 14.3 | X |  | Threats to precision |
| Intention to treat analysis used | 48 |  | X | X | X | X | X | X | 6 | 85.7 |  | X | Selection bias/attrition bias/lack of Intention to treat bias |
